# Rationally designed modular drug delivery platform based on intracellular peptide self‐assembly

**DOI:** 10.1002/EXP.20210153

**Published:** 2021-10-30

**Authors:** Hong‐Wei An, Muhetaerjiang Mamuti, Xiaofeng Wang, Haodong Yao, Man‐Di Wang, Lina Zhao, Li‐Li Li

**Affiliations:** ^1^ CAS Center for Excellence in Nanoscience, CAS Key Laboratory for Biomedical Effects of Nanomaterials and Nanosafety National Center for Nanoscience and Technology (NCNST) Beijing China; ^2^ CAS Key Laboratory for Biomedical Effects of Nanomaterials and Nanosafety, Institute of High Energy Physics Chinese Academy of Sciences (CAS) Beijing China

**Keywords:** cancer, drug delivery, molecular simulation, peptide, self‐assembly

## Abstract

Modulated molecular design‐based intracellular self‐assembly strategy has showed great potentiality in drug delivery, due to its assembling nature‐resulted optimized drug biodistribution and metabolism. The modular designing concept endows the delivery system multiple functions, such as, selectivity and universality to improve the pharmacokinetics of loaded drugs. However, the accurate controlling of the self‐assembling process in desired site to achieve optimal drug delivery is posed great challenges toward rational molecular design. Here, we fabricated a modulated drug‐delivery system (MDS) through intracellular peptide self‐assembly to realize effective drug delivery. MDS was designed based on modulated molecular designing strategy which contains five functional motifs and effectively transformed into fibrous nanostructures inside target cells by caspase3/7 hydrolysis directed in situ self‐assembly. The experimental studies and molecular simulations were carried out to evaluate the successful construction and delivering efficacy of MDS. According to the experimental results and molecular simulation analysis, the percentage of solvent‐exposed surface area of assembling modular (KLVFFAE), as well as its non‐covalent interaction between four other modules synergeticly decide the solubility of molecules. The weak intramolecular forces of the peptide back bone, such as, hydrogen bond, as well as multivalent interactions of the side chains such as, salt bridge and hydrophobic interaction both contribute to the self‐assembly of the molecules. The significant structural difference between delivering molecules optimize the system to adapt hydrophilic and hydrophobic drugs. Finally, the predicted drug delivery molecule specifically recognizes targeted cancer cell lines and self‐assembles to form fibers intracellularly, resulting in prolonged drug retention and accumulation. The regular prediction and rational molecular design will benefit the further construction and optimization of modulated drug delivery platform.

## Graphical abstract

A general modulated drug‐delivery system based on modulated design has realized effective drug delivery. According to the experimental results and theoretical calculations, the general regulations of molecule design are obtained. The regular prediction and rational molecular design will benefit the further construction and optimization of universal drug delivery platform.

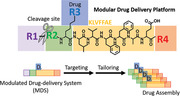

## 1 INTRODUCTION

In order to improve the pharmacokinetics of conventical therapeutic agents, the small molecular drugs can be loaded into nanocarriers through physical encapsulation or chemical conjugation to ensure its longer duration, targeted accumulation and optimal biodistribution, which result in the emergence of a new branch of medicine: The drug delivery system.^[^ ^1^ ^]^ For the past few decades, numerous efforts have been devoted to develop delivering vectors such as natural and synthetic materials including protein/peptides,^[^ ^2^ ^]^ phospholipids,^[^ ^3^ ^]^ polymers and inorganic materials,^[^ ^4^ ^]^ delivering structures including micelle, nanoparticle and nanofibers, and designing concepts.^[^ ^5^ ^]^ Although the systematic toxicity was reduced comparing with the conventional medicine, those drug delivery systems only modestly improve the therapeutic efficacy.^[^ ^6^ ^]^ More importantly, the human tumor microenvironment is remarkably abnormal, and it poses several barriers to nanomedicine delivery that cannot be overcome by the enhanced penetration and retention effect alone.^[^ ^7^ ^]^

In order to address those challenges, our group developed a strategy of in vivo self‐assembly, which combines the dynamic active targeting behavior with in situ construct desired nanostructure to deliver and accumulate drugs.^[^ ^8^ ^]^ In vivo self‐assembly is a process that exogenous small molecules self‐assembled into specific superstructure by the stimulation of the pathological abnormalities such as, over expressed enzymes,^[^ ^9^ ^]^ low pH^[^ ^10^ ^]^ and redox condition,^[^ ^11^ ^]^ fabricating highly ordered superstructures to facilitate higher therapeutic efficacy by improved penetration, accumulation/retention and systematic toxicity reduction of loaded drugs.^[^ ^12^ ^]^ Recently, our group developed a smart drug delivery system, the tumor‐selective cascade activatable self‐detained system (TCASS) to achieve the effective delivery of chemotherapeutic agents to tumor.^[^ ^13^ ^]^ Through the modulated‐molecular design strategy, the predesigned molecules circulated as monomers during the transportation. After arrived at tumor site, the molecules recognized by X‐linked inhibitor of apoptosis protein (XIAP) to activate the caspase‐3/7, then the activated enzyme cleaves the molecule to initiate its onsite self‐assembly. The TCASS drug delivery systems not only presented aggregation/assembly induced retention effect, but also presented enhanced penetration and higher drug delivery efficacy (9.2 ± 0.5% ID g^−1^, 48 h i.v. injection). Thus, modulated molecular design based in vivo self‐assembly strategy is an effective way to construct a universe drug delivery platform to obtain an optimal superstructure suitable for the delivery of different structured hydrophobic drugs.^[^ ^14^ ^]^ However, the in vivo self‐assembly is a complex process that governs by multiple factors. In addition, it is a great challenge for the rational design of molecules to ensure that the designed molecules circulate stably in monomer state, and start their rapid self‐assembly after reaching the desired site of action in complex biological systems.

**FIGURE 1:**
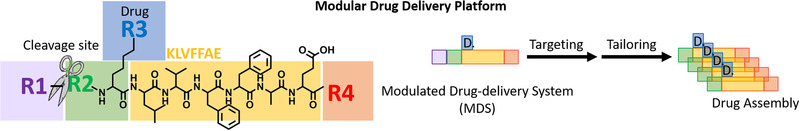
Illustration of modular designed drug delivery platform. The modular drug‐delivery system (MDS) is consisted of five different modules, including assembly module (KLVFFAE, yellow), targeting and tailoring module (purple), drug conjugated module (blue), extending modules (green and red). First, the MDS molecules were specific binding to its target through targeting module and activate the caspase3/7, then the activated caspase 3/7 tailored the MDS molecules and induced in situ self‐assembly into drug‐rich nanostructures (drug assemblies)

Herein, based on the in vivo self‐assembly nanotechnology reported by our group, we introduced rational modification and molecular simulation to give insights on molecular design pattern. We first rationally designed a modulated drug‐delivery system (MDS) through modulated molecular designing concept (Figure 1). The MDS consisted of five functional modules including assembly module (KLVFFAE, yellow), targeting and tailoring module (purple), drug conjugated module (blue), extending modules (green and red). In order to achieve its stable circulation as monomer state to arrive the pathological site and initiate its rapid self‐assembly in complex biological environment, we utilized the self‐assembly motif (KLVFFAE) as a conservative module to introduce four modification sites including R1, R2, R3, R4. The NH_2_ group on the side chain of Lys (K) was set as the drug (R3 in blue) modification site. The optimized drug delivery molecules presented as dispersed state in aqueous solution under designed concentration. After systematic administration, the molecules actively interact with its target and subsequently tailored to trigger in situ self‐assembly to enhance the localized drug concentration. In order to give insights on rational molecular design for intracellular self‐assembled nanomedicine, we carried out experimental study and molecular simulation analysis on those predesigned molecules to reveal the molecular designing pattern on the self‐assembling process. The percentage of solvent‐exposed surface area (%SASA) for assembly modular (KLVFFAE), as well as, different site modification was relevant to the solubility behavior of molecules. The optimized molecules all displayed an enzyme‐triggered intracellular self‐assembling behavior, which had high specificity for targeted tumor cell lines and can enhance drug accumulation.

## 2 RESULTS AND DISCUSSION

### 2.1 Design of modular drug‐delivery system

**FIGURE 2:**
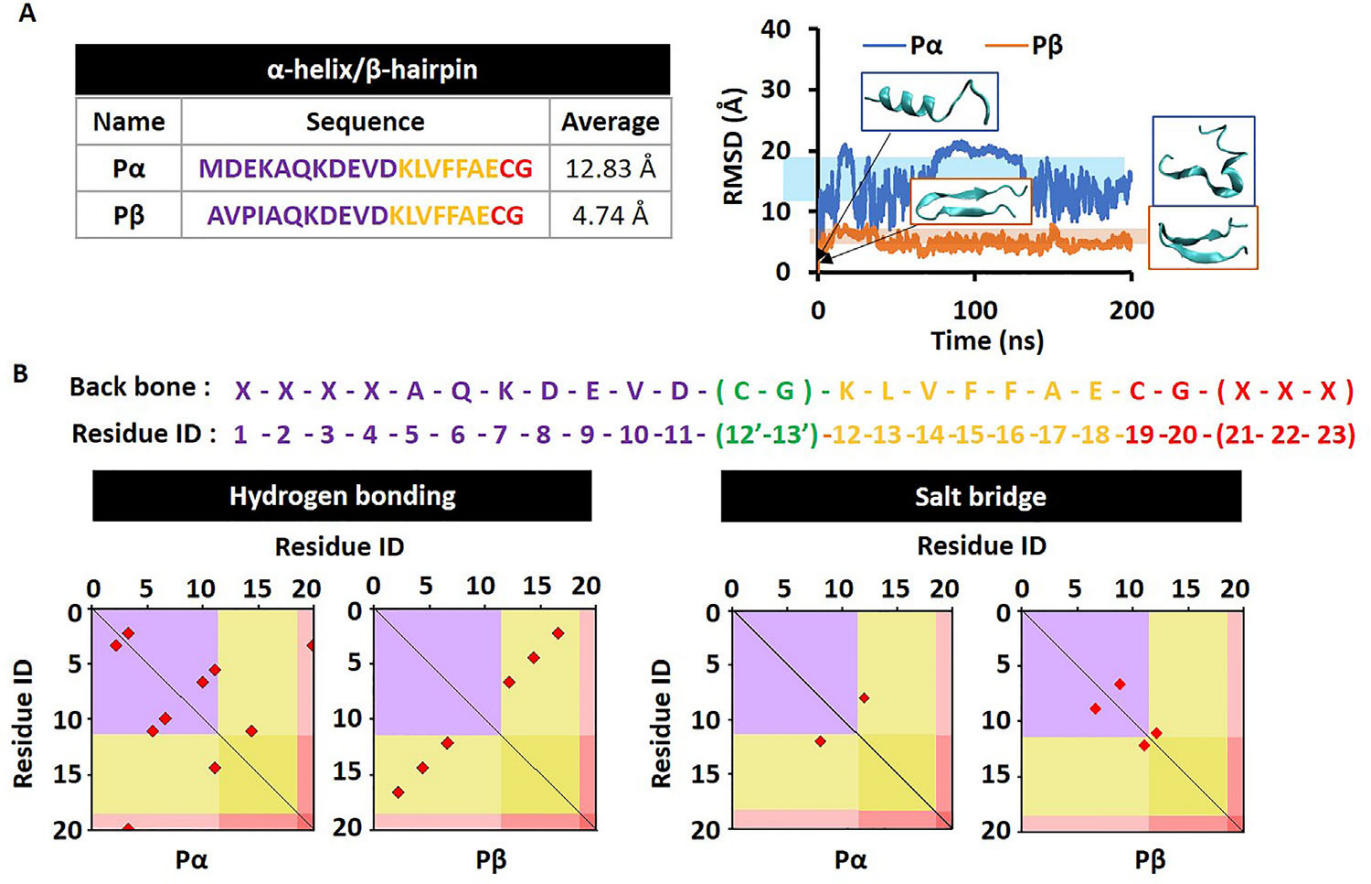
Molecular stabilities and electrostatic interactions. (A) The root‐mean‐square deviation comparison of Pα versus Pβ. (B) The distribution landscapes of electrostatic interaction modes according to module division including hydrogen bond and salt‐bridge for Pα and Pβ

For the in vivo construction of MDS, we designed eight different‐sequenced molecules through introducing different functionalized modules on four modification sites (Table 1). All the molecular characterizations were presented in Figures S1–S8. In order to initiate its efficient self‐assembly in defined location with high selectivity, we introduced a targeting and tailoring module at the N terminal of self‐assembly motif. We designed the MDS based on the typical secondary structures to confirm its basic structural stability. Furthermore, we assumed that the introduction of targeting motif by R1 domain modification directly affect the secondary structure of the designed molecules, thus, the two different targeting modules sequenced with AVPIAQK and MDEKAQK were introduced respectively. AVPIAQK is reported to bind with XIAP with high binding affinity, and after endocytosis, the specific binding of MDS with XIAP positive cell lines, lead to the activation of caspase‐3/7, which cleave the molecules to trigger the self‐assembly of the resultant product in situ. In order to evaluate the contribution of targeting motif on the drug accumulation efficacy, we mutated the conservative sequence of XIAP binding (AVPI) into MDEK. to construct a control motif sequenced as MDEKAQK. As a result, we constructed the peptide sequences with the typical secondary structures as α‐helix (denoted as Pα) and β‐sheet (denoted as Pβ) by the prediction of I‐TASSER. The molecular dynamics (MD) relaxation calculations of these two molecules with different R1 showed that Pα can hardly keep that initial α‐helix structure with averaged root‐mean‐square deviation (RMSD) as 12.83 Å, while Pβ can almost keep the β‐hairpin structure with averaged RMSD as 4.74 Å (Figure 2A). Therefore, the following delivery molecules were mainly designed based on Pβ platform with typical β‐hairpin structure.^[^ ^15^ ^]^ In addition, the hydrogen bonds and slat‐bridges were highlighted in red dots according to the residues constructing them in Pα and Pβ, respectively, in Figure 2B. The distribution landscapes of hydrogen bonds and salt‐bridges were systemically analyzed along the modular backbone distribution. The sufficient molecular interactions guarantee the structural stability of Pβ. In order to study the contribution of C terminal modification of assembly modular to molecular stability, we introduced CG into the R4 domain of Pα and Pβ, respectively, and then covalently linked the fluorescent molecule Cy, named with Pα‐R4C and Pβ‐R4C. Next, we hypothesized that the additional N and C terminal modifications of assembly modular may result in different molecular stability. Thus, we introduced GC on R2 domain and conjugated with Cy (Pβ‐R2C), to study the position effect of side modification. In addition, different motifs were introduced into the R4 domain, including fluorescent molecule fluorescein isothiocyanate FITC (termed as Pβ‐R4F) and a short peptide PDIFD (termed as Pβ‐R4P) to study the effect of R4 domain modification on molecular stability. Finally, we chose hydrophobic molecule pyrene (Py) as a demo drug modified at R3 domain of molecule Pβ‐R4C (named as Pβ‐R4C‐D) to demonstrate the feasibility of the drug delivery system.

**TABLE 1:** The designed molecules with various modules

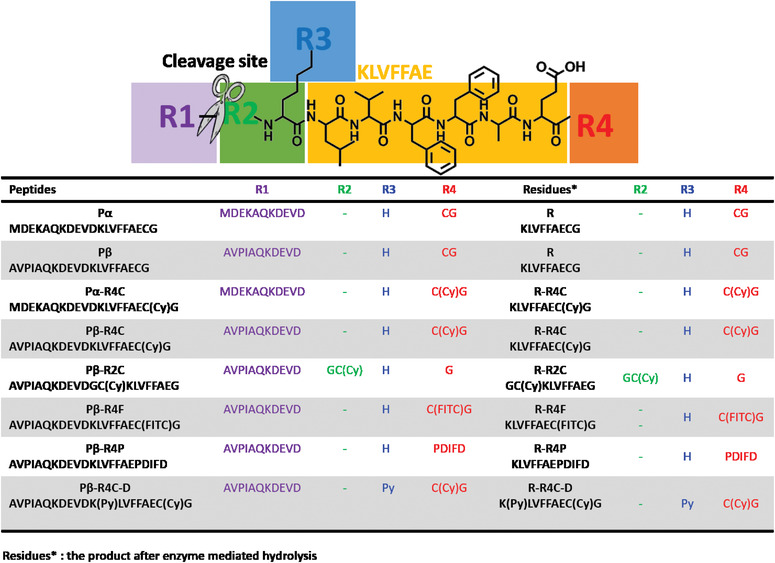

### 2.2 Self‐assembling behavior of designed molecules

**FIGURE 3:**
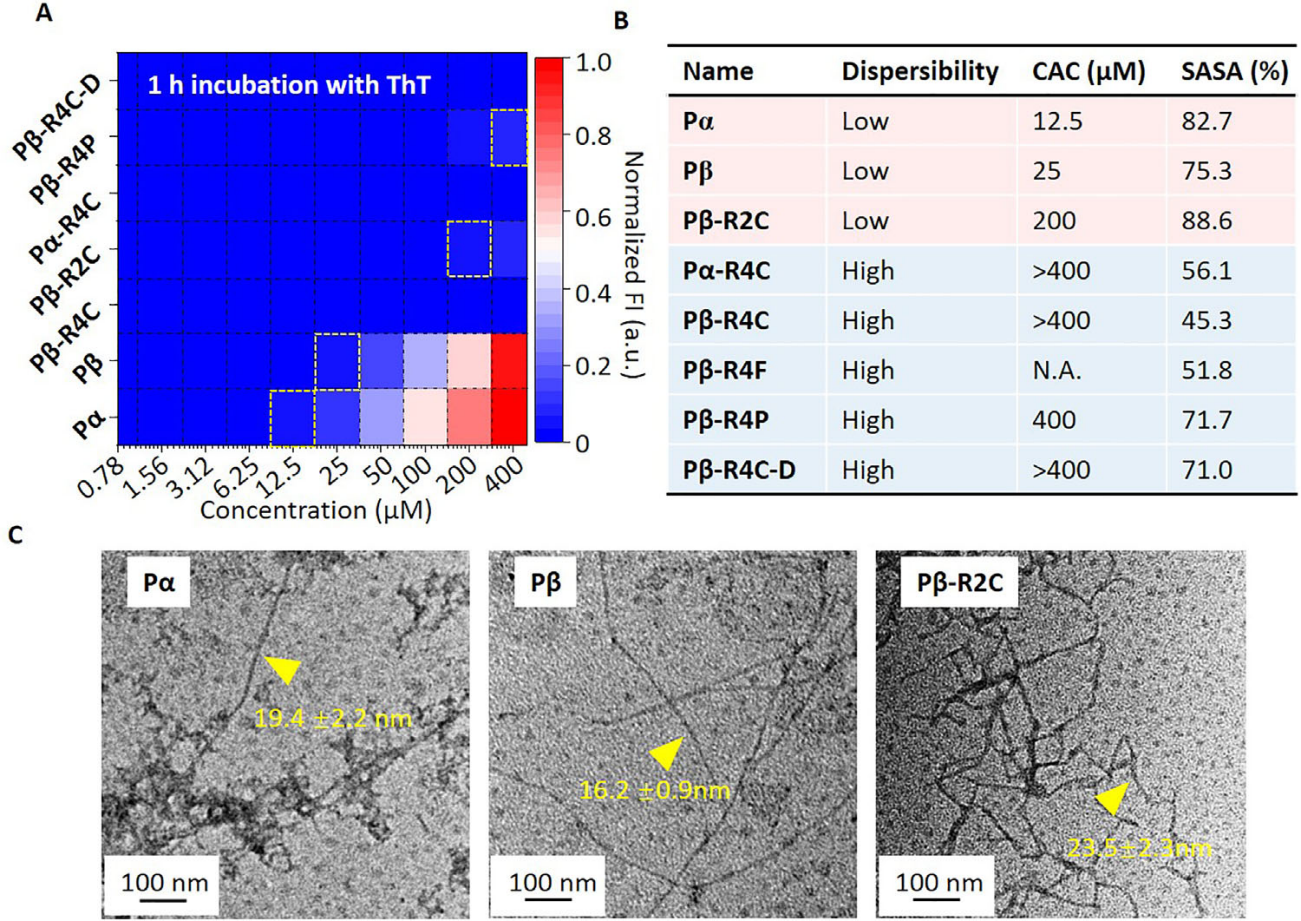
Dispersibility of designed molecules. (A) The dose dependent assembling profiles after incubation with Thioflavin T (ThT) for 1 h. (B) The table listed dispersibility, critical aggregation concentration (CAC) and percentage of solvent‐exposed surface area (%SASA) for Pα, Pβ, Pα‐R4C, Pβ‐R4C, Pβ‐R2C, Pβ‐R4F, Pβ‐R4P and Pβ‐R4C‐D. (C) The TEM images of Pα, Pβ and Pβ‐R2C nanofiber assemblies

For intracellular self‐assembly, the rapid transformation from dispersed state to aggregate state in desired site is required. In other words, the molecules need to be stable monomeric states at other locations and present self‐assembled states at the required locations. Therefore, location‐specific self‐assembly characteristics are the decisive factors for the success of molecular design. In this work, we selected KLVFFAE, a peptide derived from amyloid β‐42, which had a typical antiparallel β‐sheet structure, as the assembly module.^[^ ^16^ ^]^ Then, different modification on four domains were carried out to evaluate its dispersity and self‐assembly properties. We carried out ThT (Thioflavin T) analytic assay to evaluate whether those predesigned molecules presented relatively dispersed state. ThT is a sensitive probe to the lamellar β‐sheet structure. When exposed to the lamellar β‐sheet structure, ThT fluorescence was enhanced due to inserting into the lamellar structure.^[^ ^17^ ^]^ As shown in Figure 3A, we observed that the ThT fluorescence obviously increased for molecule Pα and Pβ under a lower molecular concentration of 12.5 and 25 μM, respectively, and relatively higher molecular concentration of 200 μM for Pβ‐R2C was obtained. On the contrary, the incubation of ThT with other molecules did not induce the fluorescence increase under relatively high molecular concentration up to 400 μM. Thus, we hypothesized that the molecule Pα, Pβ and Pβ‐R2C presented a strong tendency of self‐assembly, while others exhibited a higher molecular dispersity.

In order to validate our hypophysis and quantitively determine the solubility of those molecules, we calculated the critical assembly concentration (CAC) of those molecules based on the ThT analysis. As showed in Figure 3B, the CAC of Pα and Pβ were about 12.5 and 25 μM, respectively, which is identical with the enhanced ThT fluorescence observed in Figure 3A. However, other molecules with R4 domain modifications all presented a higher CAC with ≥400 μM. Those results indicated that the end site (R4) modification of assembling module dramatically increased the solubility of designed molecules. We also observed that the R2 domain modification also increased its CAC up to 200 μM which is lower than other molecules with R4 domain modification, which indicates that the influence of R4 modification on the tendency of molecular self‐assembly is bigger than R2 modification.

To figure out the influence of different modification on molecular solubility from the aspect of molecular interaction, we performed the MD calculations to study the solvent accessible surface area (SASA) of assembling module KLVFFAE.^[^ ^18^ ^]^ Calculation of SASA values is one of the effective approaches for predicting the molecular assembling behavior that the change of its SASA value (%SASA) after molecular relaxation is the effective chemophysical criterion in the dispersity or assembling property of peptides^[^ ^19^ ^]^ (Figure 3B). The lower soluble molecules, such as Pα, Pβ, and Pβ‐R2C, had a high solvent‐exposed surface area of KLVFFAE over 75%. It is indicated that the higher exposure of the assembled domain might increase the multiple intermolecular interaction probability to form β‐sheet assemblies. Besides, compared with Pα‐R4C and Pβ‐R4C, the Pβ‐R4C with the β‐hairpin backbone exhibited a lower %SASA than that of α‐helix structure, which may due to the hairpin structure benefitted for covering the exposure surface of assembled domain.

To further confirm our speculation, we observe the assembled morphology of these molecules under its CAC concentrations. The TEM images of low dispersibility molecules (Pα, Pβ, and Pβ‐R2C) showed nanofibrous structures (Figure 3C). As those results indicated, the modification position of fluorescence molecules largely influenced the dispersity of designed molecules. As %SASA indicated, the R2 domain modification of Cy molecules increased the %SASA up to 88.6%, the R4 domain modification, however, dramatically revised the exposure area (45.3%) and enhanced dispersibility. As a threshold, the %SASA value around 75% distinguishes between assembly and soluble states. Thus, the optimal choice for modification position is decisive in %SASA regulation for successful molecular design.

### 2.3 Multi‐interactions and secondary structures of backbone

**FIGURE 4:**
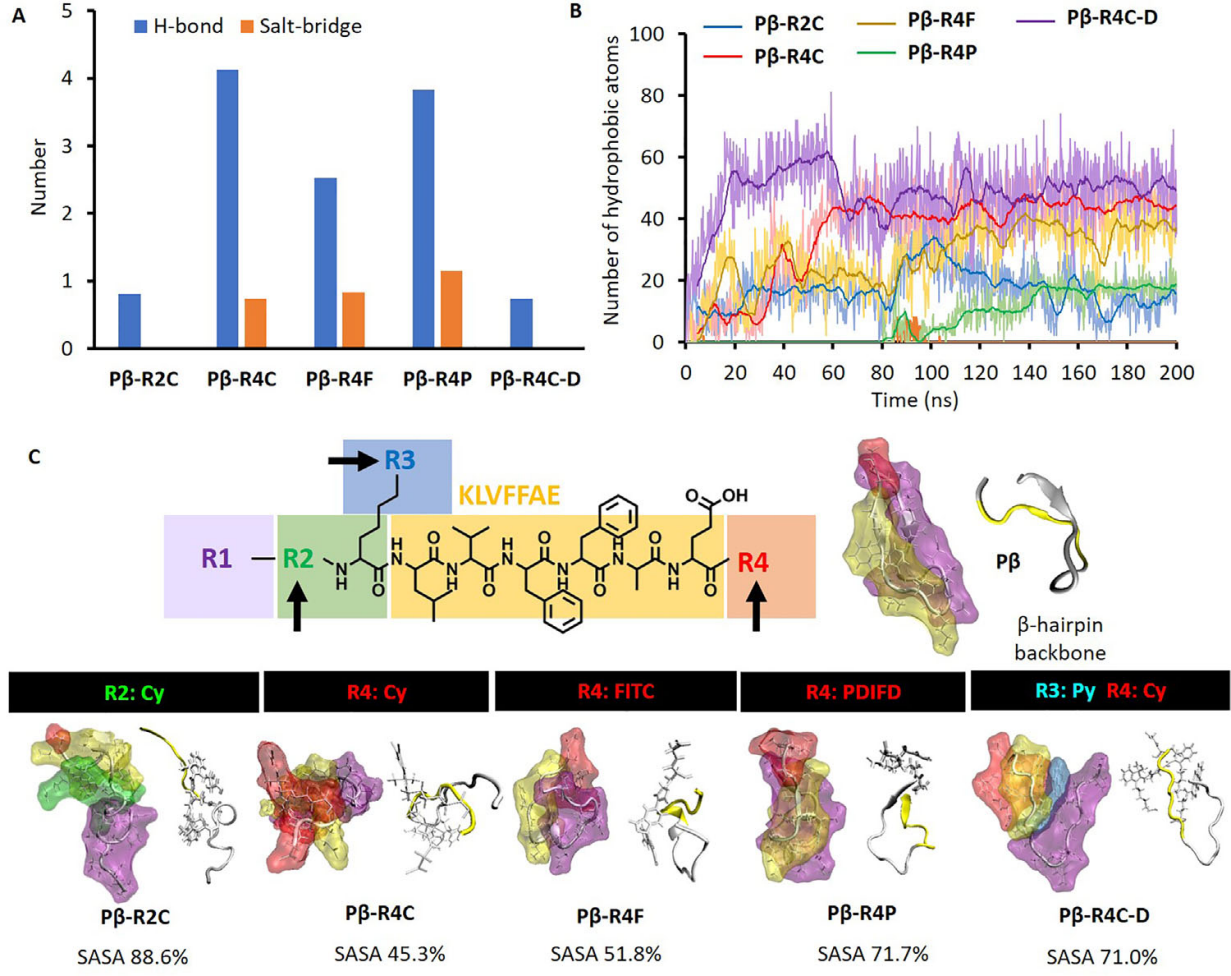
Multi‐interactions and second‐structures. (A) The averaged numbers of hydrogen bonds/salt‐bridges and (B) the total atom number of the hydrophobic interaction among multi‐modules varies with the dynamic simulation process for Pβ‐R2C, Pβ‐R4C, Pβ‐R4F, Pβ‐R4P and Pβ‐R4C‐D. (C) The relaxation molecular conformations of the designed delivery molecules based on Pβ in isosurface representation with their secondary structures of backbone for Pβ, Pβ‐R2C, Pβ‐R4C, Pβ‐R4F, Pβ‐R4P and Pβ‐R4C‐D

The present drug delivery system could be rationally designed by the %SASA regulation of assembly module. However, %SASA is a result parameter stem from the molecular multi‐interactions of entire designed molecule. The beneath molecular multi‐interactions can decide the secondary structures of backbone, additionally, can reveal the adjustment mechanism of %SASA, then provide the rational design idea for the effective drug delivery platform.^[^ ^20^ ^]^ First of all, the electrostatic interaction modes were analyzed according to module division including hydrogen bond and salt‐bridge for the designed eight molecules. The distribution landscapes of hydrogen bonds and salt‐bridges indicate their number and location distributions among the modules of designed molecules. For Pβ‐R4C with the lowest %SASA, there were abundant electrostatic interactions between different modules. These interactions between multi‐modules benefited the assembly module to decrease its %SASA, and supported the dispersity/assembly functions of the MDS after hydrolysis. Furthermore, we focused on the different modified molecules based on stable Pβ. In Figure 4A, the hydrogen bonds and salt‐bridges between multi‐modules were measured for Pβ‐R2C, Pβ‐R4C, Pβ‐R4F, Pβ‐R4P, and Pβ‐R4C‐D. The strengths of both hydrogen bond and salt‐bridge were distinguish in Pβ‐R4C with higher average values as 4.13 and 0.73, respectively, leading to its proper dispersity, which similar with the following molecules: Pβ‐R4P and Pβ‐R4F. As known, the hydrophobic interaction is another important intermolecular interaction, together with the electrostatic interactions, to stabilize the molecular conformation and cover the SASA of hydrophobic module. The atom number of hydrophobic interaction was counted (Figure 4B). For Pβ‐R4C‐D with hydrophobic drug, the significant intramolecular hydrophobic interactions contribute the molecular stability and assembly module was covered strongly, although its electrostatic interactions were weak. On the contrary, the electrostatic and hydrophobic interactions between modules were both weak in Pβ‐R2C causing its high %SASA and dispersity lacking.

The different strength and distribution of these multi‐interactions above are the origin of the molecular conformation differences. In Figure 4C, the relaxation molecular conformations were indicated for the designed delivery molecules based on Pβ in isosurface representation with their secondary structures of backbone. In these isosurface displays, it is intuitive to learn how the assembly module (KLVFFAE, in yellow) be covered by other modules under the electrostatic and hydrophobic multi‐interactions. The %SASA of Pβ‐R2C was obviously the largest in these five designed molecules because its assembling module was only covered by Cy without any other modules. In the secondary structure analysis of backbone, the shapes of molecules were much clearer to show that the cured and ringed assembly module benefited the low %SASA and proper dispersity, especially in Pβ‐R4C. To verify, we compared the radius of gyration (RGYR) for assembly modules in Pβ and Pβ‐R4C in Figure S10, and indeed found that the RGYR in Pβ‐R4C was smaller (under curled and ringed state) than that in Pβ corresponding to the better dispersity in Pβ‐R4C than that in Pβ.

### 2.4 Optimization of modules

**FIGURE 5:**
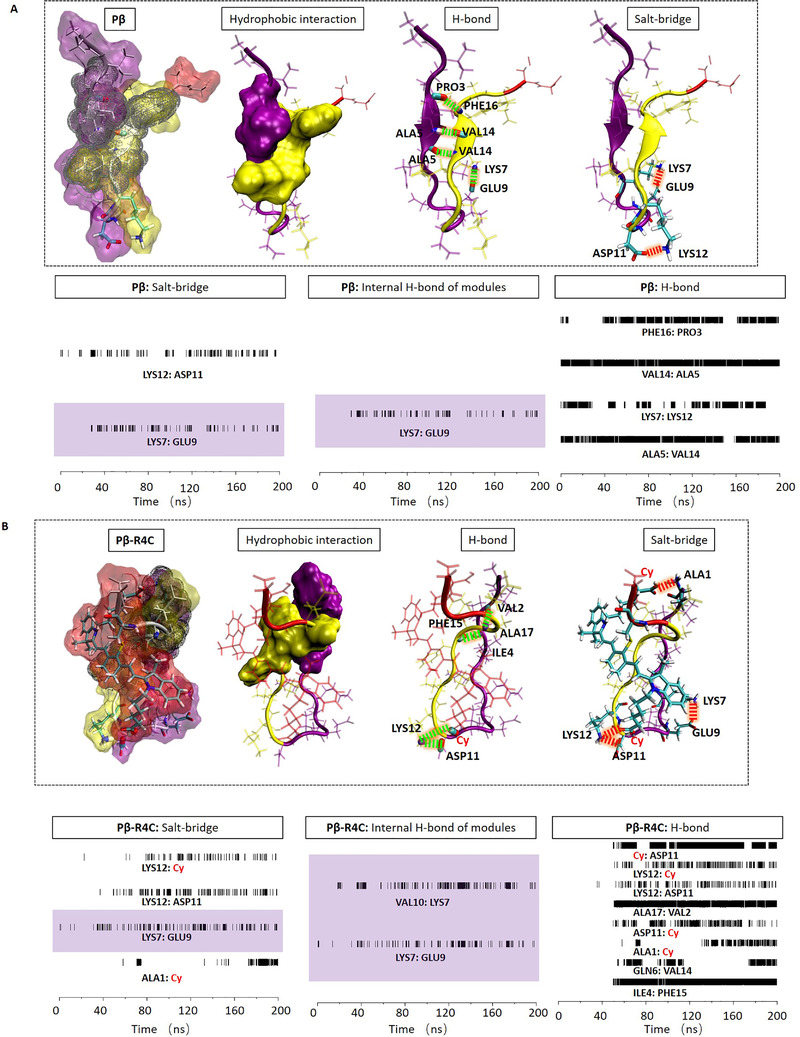
Molecular interaction details including hydrogen bond, salt‐bridge and hydrophobic, together with the lifetime analysis of hydrogen bond and salt‐bridge for (A) Pβ and (B) Pβ‐R4C

**FIGURE 6:**
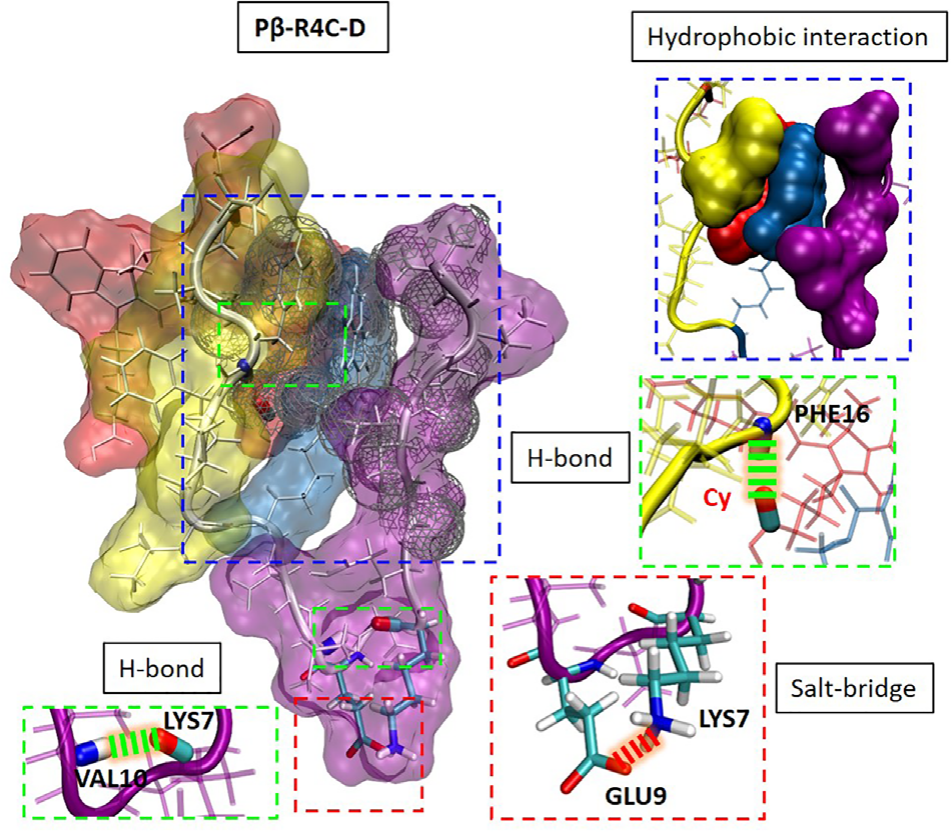
Molecular interaction details including hydrogen bond, salt‐bridge and hydrophobic for Pβ‐R4C‐D

How to optimize the modules and design the successful delivery molecules from Pβ to Pβ‐R4C, even to the drug loaded molecule as Pβ‐R4C‐D? The optimization strategy of modules was performed based on the molecular interaction details as following. First, we fully studied the molecular interaction details of Pβ as the design starting point in Figure 5A. There were four hydrogen bonds: PHE16:PRO3, VAL14:ALA5, LYS7:LYS12, ALA5:VAL14, and two salt‐bridges: LYS12:ASP11, LYS7:GLU9, as shown by the conformation with backbone. Especially in the time‐dependent analysis, four hydrogen bonds were located between different modules. At the same time, one salt‐bridge was in the same module (highlighted with color block), and the other salt‐bridge was between different modules. The inter‐module interactions mainly contribute the stability of the molecule. While, the hydrophobic interaction between R1 module and assembly module was limited as displayed in the diagram of Figure 5A. The assembly module was exposed and mostly causing aggregation. Therefore, we introduced the Cy ligand in different modules, including R2 and R4 domain in Pβ to optimize the delivery molecule as shown in Figure S11 and Figure 5B, respectively. In Figure S11 of Pβ‐R2C, the R2 module (modified with Cy) separated R1 module from assembly module, and had multiple electrostatic actions inside R1 module, but there was little electrostatic effect between modules, even between R1 module and assembly module. The exposed assembly module evoked the molecular aggregation. Therefore, the modification in R2 module was excluded in the molecular design. On the contrary, the Cy modification of R4 module introduced a lot of electrostatic interactions between modules, but rarely and electrostatic interactions within modules. In details, there were six inter‐module hydrogen bonds: Cy:ASP11, LYS12:Cy, ALA17:VAL2, ALA1:Cy, GLN6:VAL14, ILE4:PHE15; and three inter‐module salt‐bridges: LYS12:Cy, LYS12:ASP11 and ALA1:Cy, and only one intra‐module salt‐bridge: LYS7:GLU9. The abundant inter‐module interactions supported the full contact between assembly module and other modules. Especially, the R1 module provided significant hydrophobic contact on the assembly module as shown in Figure 5B. This hydrophobic interaction of R1 module not only reduced the %SASA of assembly module for better dispersity, but also facilitated the aggregation of assembly module after chemical hydrolysis (residues without R1 module). Furthermore, we tested other ligands, such as FITC and PDIFD in Pβ‐R4F and Pβ‐R4P, respectively (Figures S12 and S13). The inter‐module interactions were both weaker than that of Pβ‐R4C, resulting in higher %SASA than that of Pβ‐R4C. Therefore, after optimization design, it is regarded that Pβ‐R4C can be seen as an excellent delivery platform. Finally, we introduced Py as demo drug into Pβ‐R4C to construct a drug delivery molecule named Pβ‐R4C‐D. Details of intermolecular interactions were shown in Figure S14. As mentioned above, Py drug in R2 module could separate R1 module from assembly module, and reduce the inter‐module electrostatic interactions (Figure 6). But the hydrophobic drug not only replaced the hydrophobic residues of R1 module, but also covered the assembly module as a new hydrophobic core to stabilize the molecular conformation. The total hydrophobic interactions of the assembly module were shown in Figure 6. Obviously, it was necessary to find that Py drug interacted closely with all other modules. The results showed that the successful drug delivery molecule of Pβ‐R4C‐D can regulated the %SASA and realize the dispersity/assembly dynamic procedure.

### 2.5 The self‐assembling behavior of modulated drug‐delivery system

**FIGURE 7:**
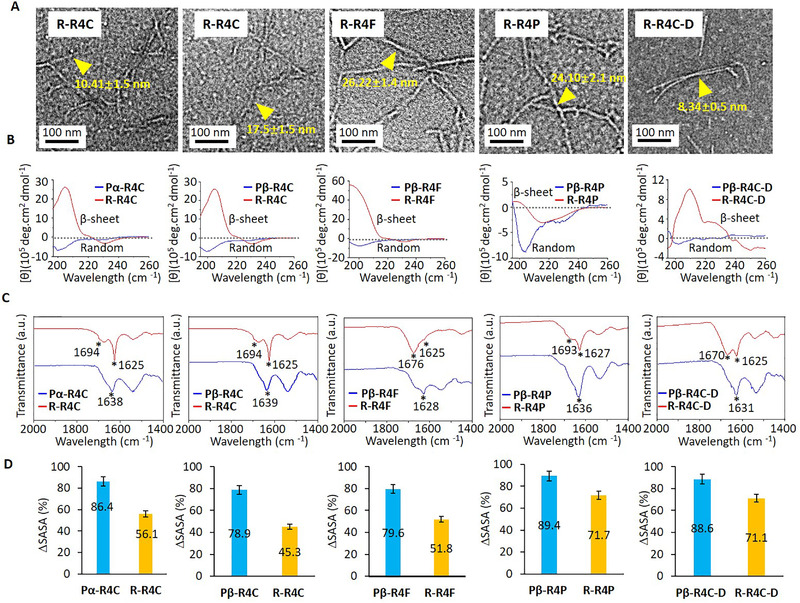
Self‐assembling behavior of drug delivery system. (A) Self‐assembling morphology of R‐R4C, R‐R4F, R‐R4P and R‐R4C‐D obtained by TEM imaging. (The same final product was obtained after Pα‐R4C and Pβ‐R4C cleavage, thus, two TEM images of R‐R4C is corresponded to the morphology of R‐R4C obtained by Pα‐R4C, Pβ‐R4C cleavage.) Nanofibers were indicated with yellow triangles. (B) The CD spectra of predesigned molecules (Pβ‐R4C, Pα‐R4C, Pβ‐R4F, Pβ‐R4P, Pβ‐R4C‐D) and its assembling residues (R‐R4C, R‐R4F, R‐R4P, and R‐R4C‐D). (C) FTIR spectra of predesigned molecules (Pβ‐R4C, Pα‐R4C, Pβ‐R4F, Pβ‐R4P, Pβ‐R4C‐D) and its assembling residues (R‐R4C, R‐R4F, R‐R4P, and R‐R4C‐D). (D) The calculated %SASA of predesigned molecules (Pβ‐R4C, Pα‐R4C, Pβ‐R4F, Pβ‐R4P, Pβ‐R4C‐D) and its assembling residues (R‐R4C, R‐R4F, R‐R4P, and R‐R4C‐D)

In order to achieve a highly ordered superstructure in vivo, it is necessary to transfer the precursor with higher CAC content to the monomer with lower CAC content at the desired position under the trigger of some chemical reactions. In this work, the pre‐designed molecules trigger the conversion of precursors to monomers transformation through enzyme‐directed hydrolysis, and the substrate (DEVD) was cleaved by caspase 3/7. Due to the increased hydrophobicity and conformational changes, the resultant product further self‐assembled into the required superstructure. The previous study in this work confirmed five molecules with higher dispersity (Pβ‐R4C, Pα‐R4C, Pβ‐R4F, Pβ‐R4P, Pβ‐R4C‐D). Here, we studied the self‐assembly properties of five related residues of these highly soluble molecules (R‐R4C, R‐R4F, R‐R4P, R‐R4C‐D).^[^ ^21^ ^]^ In order to confirm whether those molecules self‐assembled into ordered superstructures, first we directly observed its assembled morphologies through TEM imaging (Figure 7A). As showed in TEM images, all five molecules self‐assembled into nanofibers with different shapes and diameters. Then, the secondary structure of designed molecules and residues were further confirmed by CD and FTIR spectra. As indicated in CD spectra (Figure 7B), the five molecules presented random coil characteristics with negative peaks around 200 nm, while, as for the related residues, the random characteristic vanished and emerged typical β‐sheet characteristics with positive peak at 200 nm and negative peak at 230 nm. Interestingly, for molecule R‐R4C‐D, the β‐sheet characteristic positive peak at 200 nm shifted to 210 nm and negative peak at 230 nm shifted to 250 nm, which may be due to the influence of loaded drug Py on the secondary structure. Those results indicated that the chemical hydrolysis of those molecules triggered the random coil to β‐sheet structure transformation, which further promoted their self‐assembly and orienting nanofibers. As shown in Figure 7C, the FTIR results were identical with CD spectra for the hydrolyzed residues. In FTIR spectra, we observed a random coil characteristic peak around 1640 cm^−1^ for pre‐hydrolyzed molecules, while, a peak around 1620–1630 cm^−1^ were observed for the hydrolyzed residues, which indicated the β‐sheet structure.^[^ ^22^ ^]^ Due to the strong intermolecular hydrogen bond, the β‐sheet characteristic peaks shifted to a lower wavenumber below 1630 cm^−1^ which was very common in fibril structures. For molecules R‐R4C, R‐R4P, we also observed a peak around 1680–1690 cm^−1^, which indicated that the antiparallel molecular β‐sheet structure produced by the transition dipole coupling contributes to the ordered arrangement of molecules.^[^ ^23^ ^]^ Based on the FTIR results we speculated that the molecules R‐R4F, R‐R4C‐D packed with parallel β‐sheet structure duo to absent the peak near 1680–1690 cm^−1^. All the results above revealed that the molecular hydrolysis triggered the random coil to β‐sheet structure transformation, which further promoted the formation of highly ordered structure.

In order to deepen our understanding of the self‐assembly on the basis of molecules and atoms, we carried out MD calculations to reveal the difference of exposure area before and after chemical hydrolysis. As shown in Figure 7D, after hydrolysis, the %SASA was significantly increased. Because the interactions between R1 and assembly module were broken after chemical hydrolysis, the covered area of self‐assembly motifs by R1 were exposed, which further contributed to its rapid self‐assembly. All those results indicated that the hydrolysis of molecules was conducive to the highly efficient self‐assembly.

### 2.6 Specific intracellular drug delivery

**FIGURE 8:**
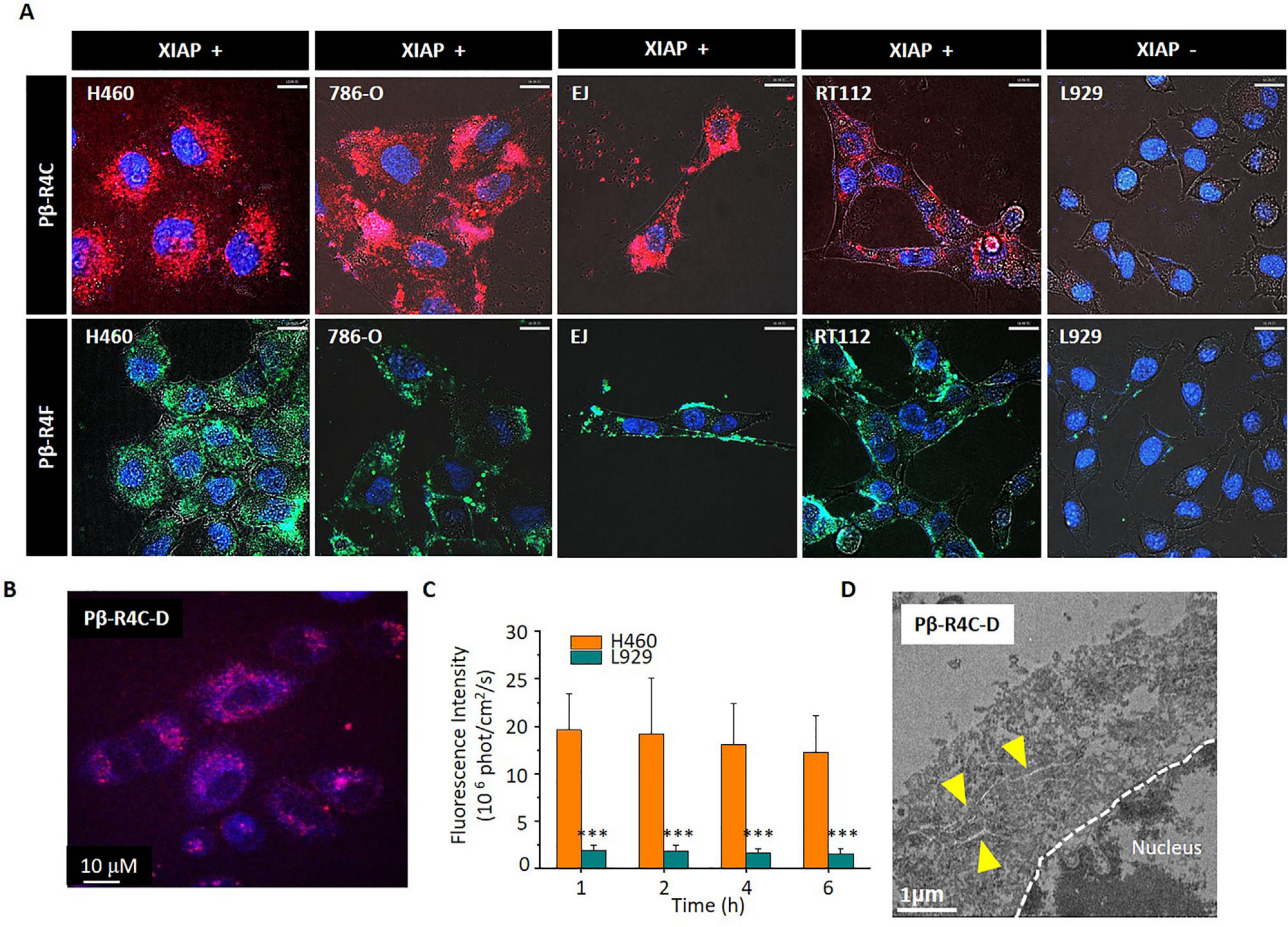
The feasibility of designed drug delivery system. (A) CLSM images of XIAP positive (H460, 786‐O, EJ, RT112) and negative cell lines (L929) coincubated with Pβ‐R4C, Pβ‐R4F, respectively. (Blue: Nucleus fluorescence, red: Cy fluorescence, green: FITC fluorescence). (B) CLSM images of H460 co‐incubated with Pβ‐R4C‐D, blue for Py and red for Cy. (C) Time‐dependent fluorescence intensity calculation of H460 and L929 coincubated with Pβ‐R4C‐D. (D) Bio‐TEM images of H460 cell section. Resultant nanofibers were indicated with yellow triangle

Drug delivery efficacy is one of the decisive factors for evaluating the drug delivery systems. Thus, in this section, we demonstrated its feasibility on serval cell lines (Figure 8A). In order to demonstrate its selectivity and universality, we chose several positive cell lines including H460, 786‐O, EJ, RT112 and XIAP negative cell line including L929. As indicated in Figure 8A, for XIAP positive cell lines,^[^ ^24^ ^]^ we observed a strong Cy and FITC fluorescent signal from molecule Pβ‐R4C and Pβ‐R4F. No obvious fluorescent signal was observed from Pβ‐R4C or Pβ‐R4F for XIAP negative cell lines, indicating that the drug delivery system had a desired selectivity and universality

In order to evaluate the drug delivery efficacy, we directly observed the Py fluorescence by CLSM imaging. As showed in Figure 8B and Figure S15, strong Py fluorescence signals appeared in XIAP positive cell lines, indicating that caspase 3/7 was activated and further cleaved the molecules inducing assembly after XIAP recognition, which was helpful for further accumulation of drug molecules. In order to systematically demonstrated the favorable drug delivery efficacy, we calculated the time dependent fluorescence signal of Py, which indirectly reflected the time dependent drug accumulation profile. As can be seen in Figure 8C, compared to XIAP negative cell line, the XIAP positive cell line presented 8‐fold increased the drug accumulation at 6 h incubation, indicating that the drug delivery system offered a durable drug. In addition, we performed the cell section analysis and directly observed the morphology of assemblies intracellularly (Figure 8D). The intracellular formed nanofibers confirmed the controllable in situ assembly of MDS. All those results verified that the drug delivery system was feasible for highly efficient drug delivery.

## 3 CONCLUSIONS

In conclusion, a modular molecule design based intracellular self‐assembly strategy was utilized to fabricate a universal drug delivery platform. The experimental studies and molecular simulations were carried out to evaluate its feasibility. The experimental results and theoretical calculations indicated that the modification site of dye molecules play a decisive role in the dispersity of predesigned molecules. Our experimental results including ThT assay, CD and FTIR analysis showed that the R4 modification hydrophilic molecule contributed to its dispersity and increase its CAC up to 400 μM. While, the R2 modification has little effect on its dispersity, and the increase of CAC was only 200 μM. The molecular analysis included electrostatic/hydrophobic interactions, secondary structure of backbone, the RGYR of assembly modules and %SASA. Both the abundant electrostatic and hydrophobic interactions between different modules, especially between R1 and assembly module, can reduce the %SASA of assembly module, and supported the dispersity/assembly functions of the delivery platform by chemical hydrolysis. Therefore, the delivery platform was designed as Pβ‐R4C rather than Pβ‐R2C. In addition, the modification of drug molecules on Pβ‐R4C (named as Pβ‐R4C‐D) introduced the significant hydrophobic interactions contributing the molecular stability and assembly module covering, and presented little difference on its influence that under the catalysis of enzyme. The further self‐assembled into fibrous structure of molecular residues can effectively deliver drugs into desired cells with high selectivity. The development of modular molecular design strategy and the prediction of assembly mode will contribute to the construction and optimization of general drug delivery system.

## CONFLICT OF INTEREST

The authors declare no competing financial interest.

## Supporting information



SUPPORTING INFORMATION
