# Supplementary material for: Rationally designed modular drug delivery platform based on intracellular peptide self‐assembly
Source: Exploration (Beijing). 2021 Oct 30;1(2):20210153. doi: 10.1002/EXP.20210153 (PMC10190849; doi:10.1002/EXP.20210153)
Supplement: Supplementary file 1 — SUPPORTING INFORMATION [file EXP2-1-20210153-s001.docx]

Supporting information

Rationally Designed Modular Drug Delivery Platform Based on Intracellular Peptide Self-Assembly

Hong-Wei An^1#^, Muhetaerjiang Mamuti^1#^, Xiaofeng Wang^2^, Haodong Yao^2^, Man-Di Wang^1^, Lina Zhao^2,^ *, Li-Li Li^1,^ *

KEYWORDS: Peptide, Self-Assembly, Drug delivery, Molecular Simulation, Cancer

*^1^ CAS Center for Excellence in Nanoscience, CAS Key Laboratory for Biomedical Effects of Nanomaterials and Nanosafety, National Center for Nanoscience and Technology (NCNST), No. 11 Beiyitiao, Zhongguancun, 100190 Beijing, China;*

*^2^ CAS Key Laboratory for Biomedical Effects of Nanomaterials and Nanosafety, Institute of High Energy Physics, Chinese Academy of Sciences (CAS), No. 19B Yuquan Road, 100049 Beijing, China;*

*Corresponding authors:*

*Li-Li Li: orcid.org/0000-0002-9793-3995; Email:* [*lill@nanoctr.cn*](mailto:lill@nanoctr.cn)

*Lina Zhao: orcid.org/0000-0002-9796-0221; Email: linazhao@ihep.ac.cn*

1. **Material and characterization**

**Materials:** All the 9-Fluorenylmethoxycarbonyl (Fmoc)–protected amino acids were purchased from GL Biochem (Shanghai, China). The fluorescence molecules Fluorescein isothiocyanate (FITC) and model drug pyrene (Py) were purchased from Sigma Aldrich. The fluorescent dye Cy were synthetized based on our previous work^[1]^. H460, 786-O, EJ, RT112, L929 Cell lines were purchased from National Infrastructure of Cell Line Resource.

**Molecular synthesis**: standard Fmoc solid-phase peptide synthesis method was utilized for peptide synthesis followed with conjugation of fluorescence molecules Cy and FITC in PBS solution. Briefly, the Gly resin (loading: 0.37 mM/g) is utilized as the phase supports, then repeated deprotection of Fmoc resides (piperidine: 20% v/v) and acrylation (NMM: 4%, HBTU: 6%) in anhydrous DMF. For the synthesis of **Pβ-R4C-D**, after completing the coupling of last amino acid, the Fmoc group on lysine was deprotected, the py is dissolved in DMF (NMM: 4%) and further conjugated to the peptide overnight. For achieving the final product, the peptide is cleaved from resin with the mixture of TFA, TIPS and H_2_O in the volume ratio of 95: 2.5:2.5 for 2.5 h. The final solution is concentrated, precipitated in ether and purified by reverse-phase high-performance liquid chromatography (HPLC). The fluorescence molecules FITC and Cy labeling were carried out in PBS solution for 2 h in room temperature. The fluorescent molecule labeled final product was purified by reverse-phase high-performance liquid chromatography (HPLC).

**ThT assay:** the ThT assay was utilized to calculate the critical assembling concentration. Frist, the predesigned molecules with different concentration were aged for 1 h in aqueous solution, then coincubated with 20 uM ThT for 15 min. Next, the fluorescence of ThT was measured with the excitation wavelength with 450 nm. Finally, the fluorescence intensity was recorded and normalized.

**TEM imaging:** Transmission electron microscopy (TEM, Tecnai G2 20 S-TWIN) is operated at an accelerating voltage of 200 keV to observe the morphology and diameters of peptide molecules. For **Pα** and **Pβ,** 100 uM **Pα** and **Pβ**, were aged for 1 h, and the solution droplets were placed on copper grids for 5 min following with staining by uranyl acetate for 2 min. For **R-R4C**, **R-R4F**, **R-R4P**, **R-R4C-D**, the molecules were dissolved in aqueous solution containing 10% acetonitrile and 0.1% TFA, and aged for 1 h, then the solution droplets were placed on copper grids for 5 min following with staining by uranyl acetate for 2 min.

**The secondary structure analysis:** Circular dichroism (CD) spectra and Fourier transform infrared (FTIR) were utilized for analyzing the secondary structure of peptide molecules. The CD spectra were obtained by using a J 1500 CD spectrometer (Jasco, Japan), with a scanning speed of 500 nm min^-1^ and a resolution of 0.5 nm. For FTIR analysis, the samples were aged for 1 h, and 100 μL solution was spun down at 10000 g for 30 min, and the pellet was blown dried with nitrogen. The dry sample was mixed with dehydrated KBr crystals at a ratio of 5:95 (w/w), pressed into a KBr/peptide pellet, and analyzed on a MAGNA-IR 560.

**Cell culturing and CLSM imaging:** H460, 786-O, L929 cell lines and EJ, RT112 cell lines were cultured in PRMI-1640 and DMEM respectively with 10% (v: v) fetal bovine serum, 1% penicillin–streptomycin in an incubator (Thermo Scientific) at 37 °C with 5% CO_2_ and 95% relative humidity. For observing the selectivity of MDS by CLSM imaging, all the cell lines were seeded with 1 x 10^5^ cells per well, and cultured for 24 h, the cells were incubated with 50 μM **Pβ-R4C** and **Pβ-R4F** respectively at 37 °C for 1 h and followed by PBS washing. Subsequently, the cells were stained with Hoechst 33342 (1 mg ml^-1^) at 37 °C for 10 min. Finally, the cells were washed by PBS twice and immediately observed under confocal laser scanning microscopy. For observing the selective drug accumulation by CLSM imaging, the H460 cell and L929 cells were seeded with 1 x 10^5^ cells per well, and cultured for 24 h, then cells were incubated with 50 μM **Pβ-R4C-D** for 1 h, followed by PBS washing and observed by CLSM.

**Simulation Method**

The GROMACS software package^[2]^ (version 5.1.2) was used to perform energy minimization and molecular dynamics simulations using the AMBER99SB-ILDN^[3]^ force field. Structures were solvated in a box of TIP3P water model^[4]^ , and then ionized and neutralized with Na^+^ and Cl^−^ ions to a 0.15 mol/L molar concentration. Periodic boundary conditions (PBC) were set for all directions. After energy minimization, each structure undergoes NVT equilibration and NPT production. The Nose-Hoover thermostat^[5]^ was used to maintaining the system’s temperature of 300K and the Parrinello−Rahman algorithm^[6]^ was used to maintaining the pressure at 1 atm. Water molecules were constrained by the SETTLE algorithm^[7]^. The cutoff distance of van der Waals (vdW) interactions was 12 Å, and the electrostatic interactions were calculated by the particle mesh Ewald (PME) method^[8]^. All solute hydrogen bonds were constrained to their equilibrium values using the LINCS algorithm^[9]^. The timestep was 2 ps in production runs, and the coordinates were saved every 100 ps. Totally time of each system was 200 ns. The initial structure of Pα and Pβ are constructed by I-TASSER^[10]^, which are α-helix and β-sheet structures, respectively. In order to confirm the stable secondary structure of Pβ, we used the initial straight conformation of amino acid sequence to relax the Pβ and also obtained the typical β-sheet structure (Figure S9). Accordingly, the initial structure of other amino acid sequence composed of different modules were relaxed starting from straight conformations for the sufficient conformation search. The results were analyzed after relaxation to stabilize the structure. The VMD^[11]^ software was used to observe and draw the image of the structures.

1. **Scheme and figures**

**Pβ**, **Pα**, **Pα-R4C**, **Pβ-R2C** and **R-R4C** was Synthesis and characterization in previously published articles (*Nat. Commun.* **2019,** *10* (1), 4861).

**```````
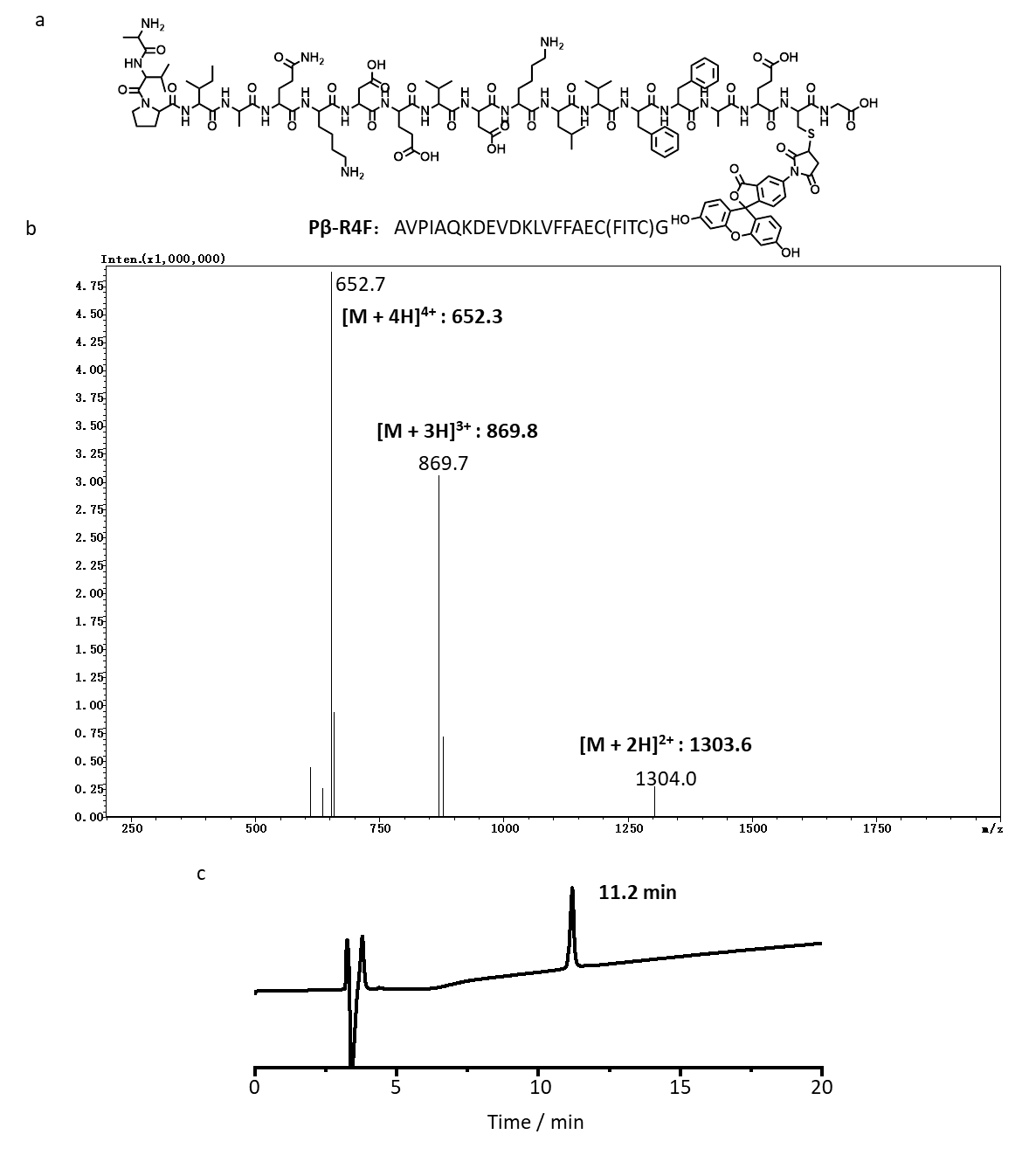
**

**Figure S1.** (a) Chemical structure of **Pβ-R4F**. (b) Electrospray Ionization (ESI) mass spectrometry analysis of **Pβ-R4F**. (c) HPLC analysis of **Pβ-R4F**.

**
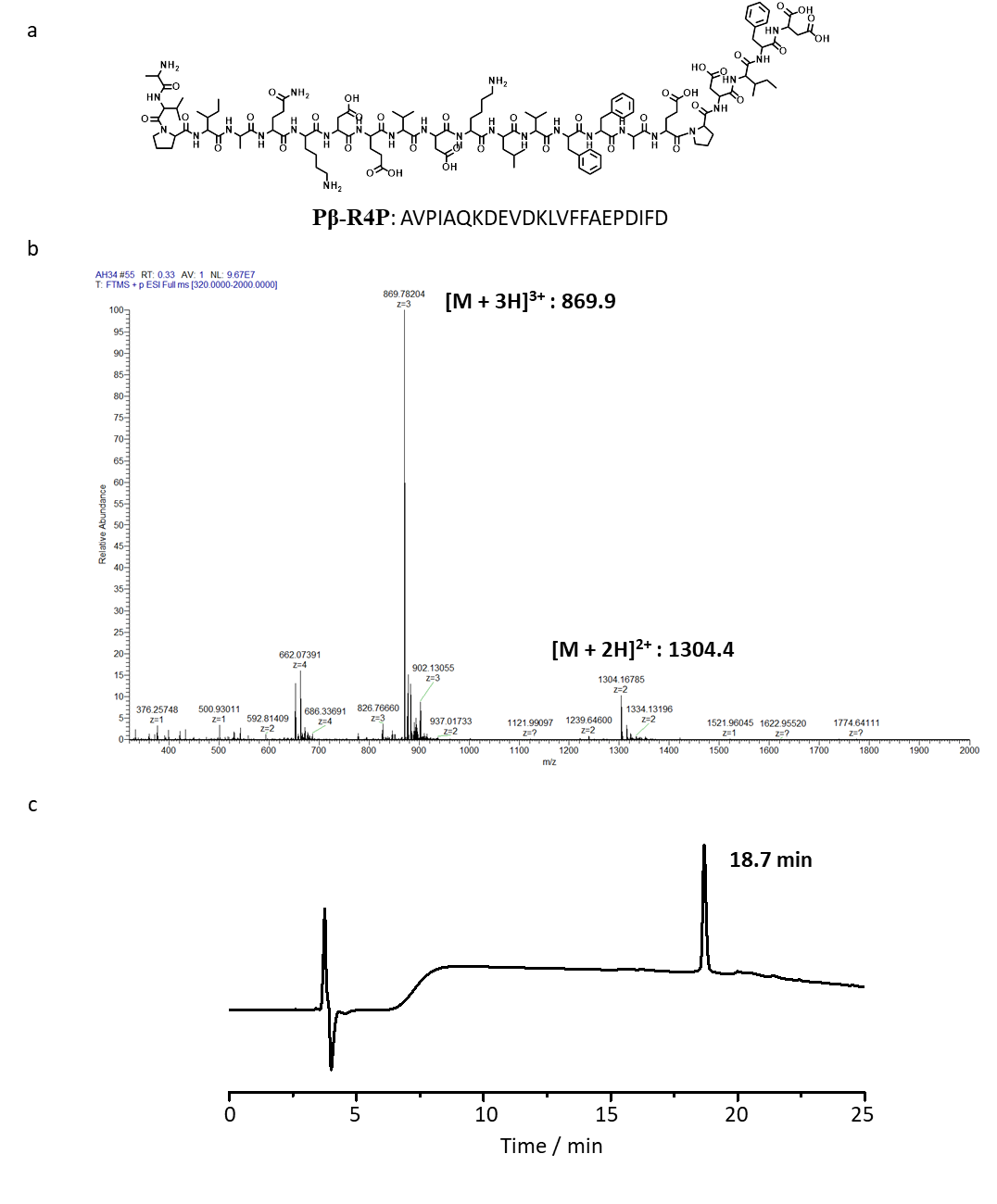
**

**Figure S2.** (a) Chemical structure of **Pβ-R4P**. (b) Electrospray Ionization (ESI) mass spectrometry analysis of **Pβ-R4P**. (c) HPLC analysis of **Pβ-R4P.**

**
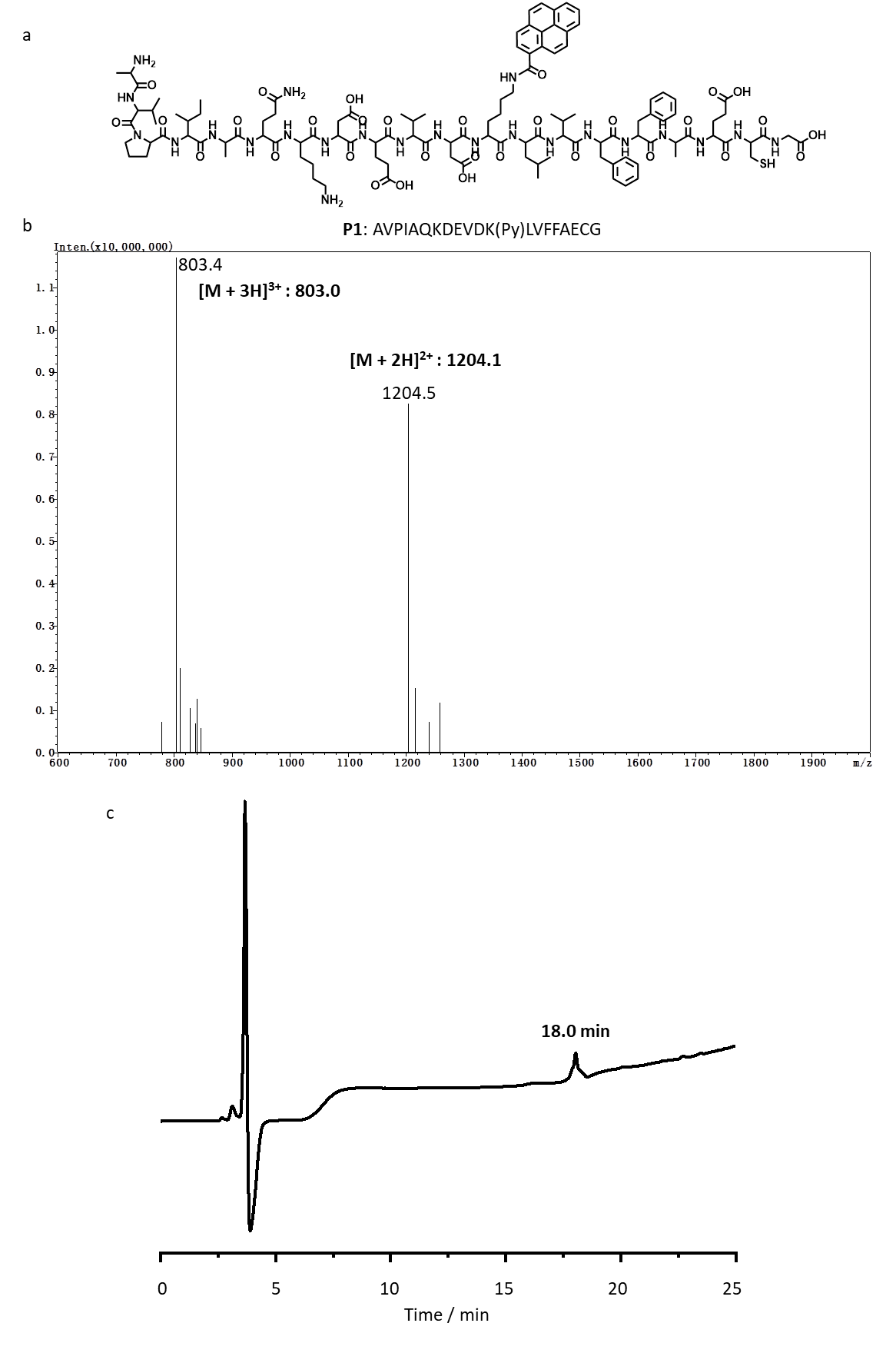
**

**Figure S3**. (a) Chemical structure of **P1**. (b) Electrospray Ionization (ESI) mass spectrometry analysis of **P1**. (c) HPLC analysis of **P1**.

**
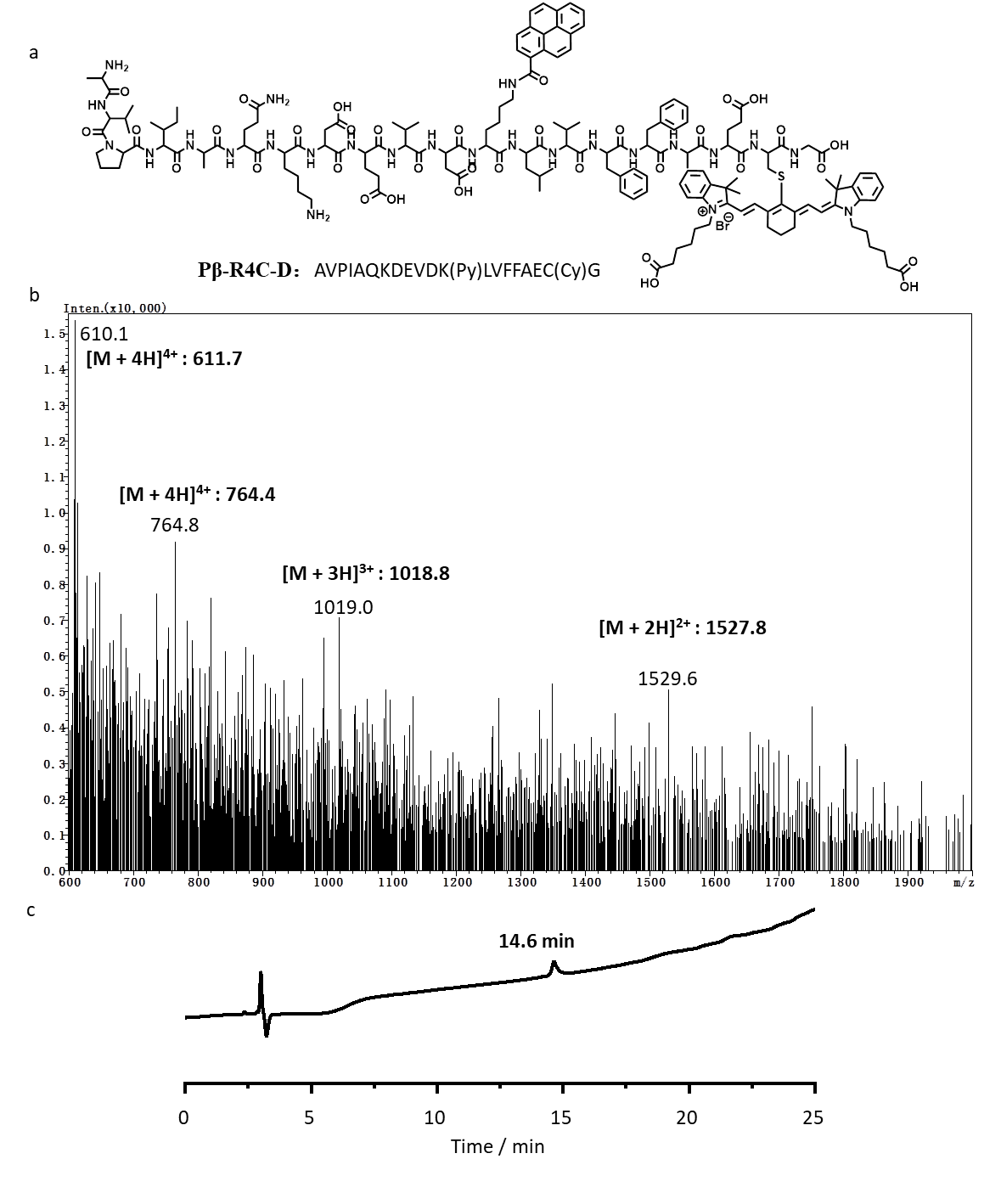
**

**Figure S4.** (a) Chemical structure of **Pβ-R4C-D**. (b) Electrospray Ionization (ESI) mass spectrometry analysis of **Pβ-R4C-D**. (c) HPLC analysis of **Pβ-R4C-D**.

**
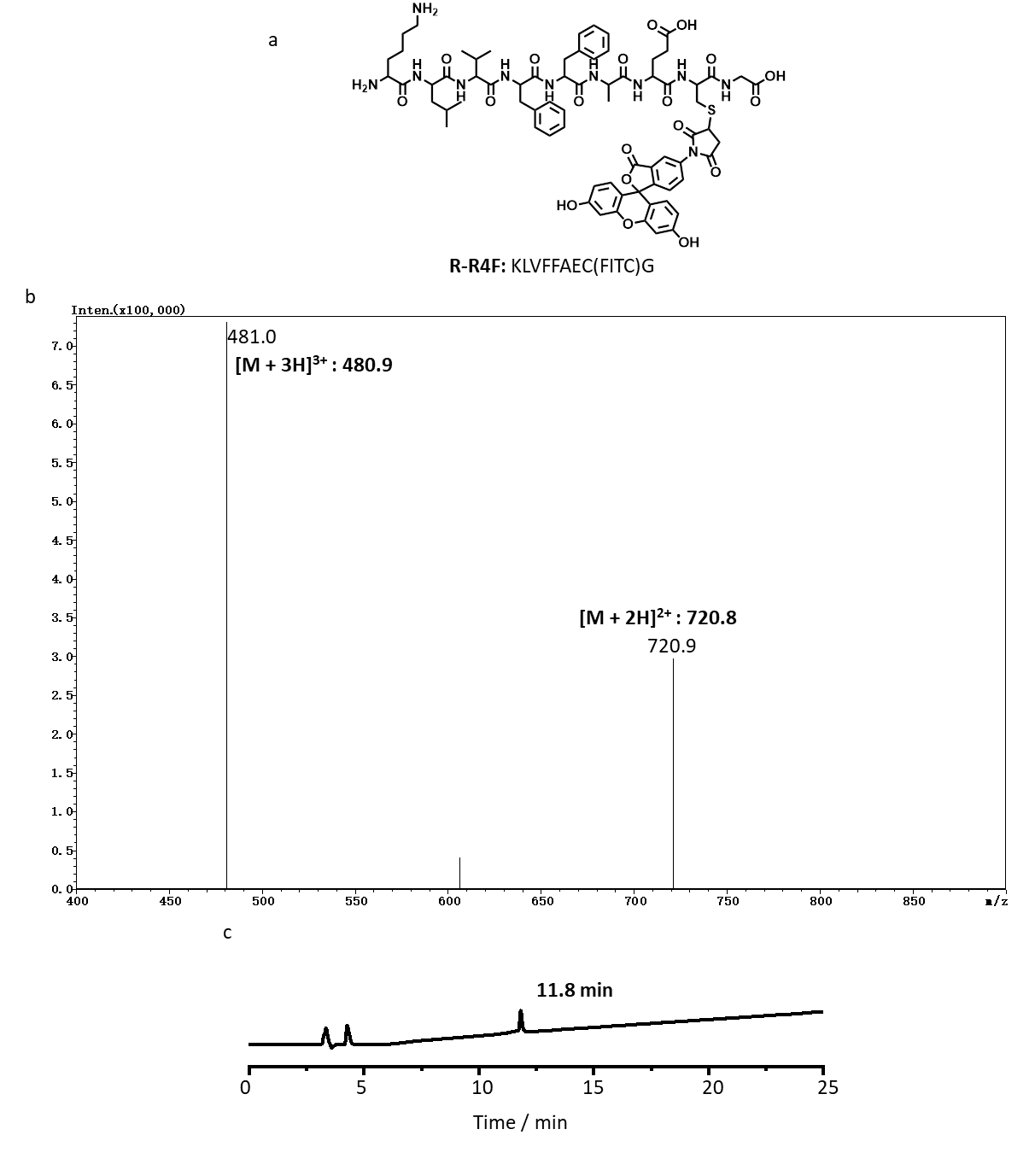
**

**Figure S5.** (a) Chemical structure of **R-R4F**. (b) Electrospray Ionization (ESI) mass spectrometry analysis of **R-R4F**. (c) HPLC analysis of **R-R4F.**

**
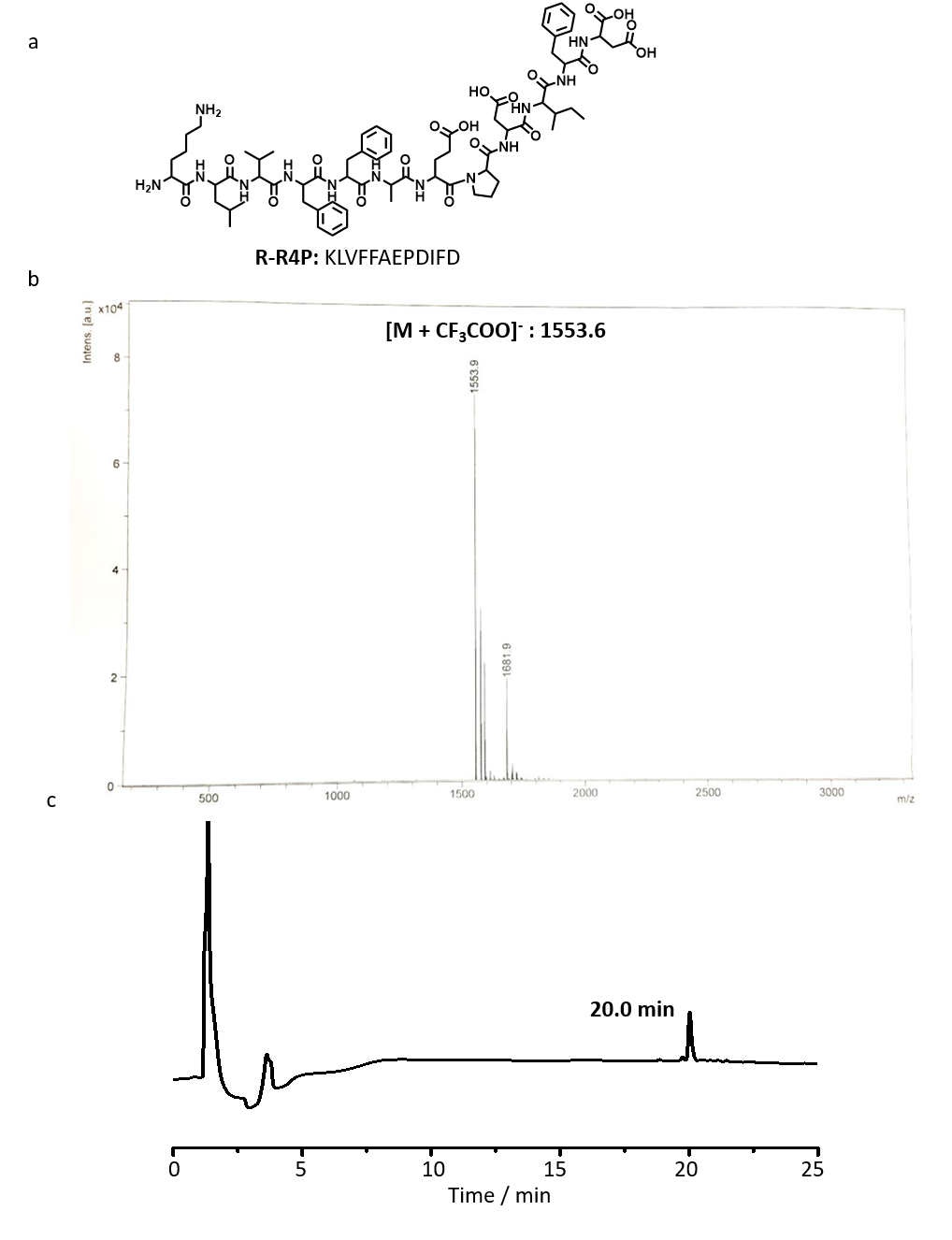
**

**Figure S6.** (a) Chemical structure of **R-R4P**. (b) MALDI-TOF-MS analysis of **R-R4P**. (c) HPLC analysis of **R-R4P.**

**
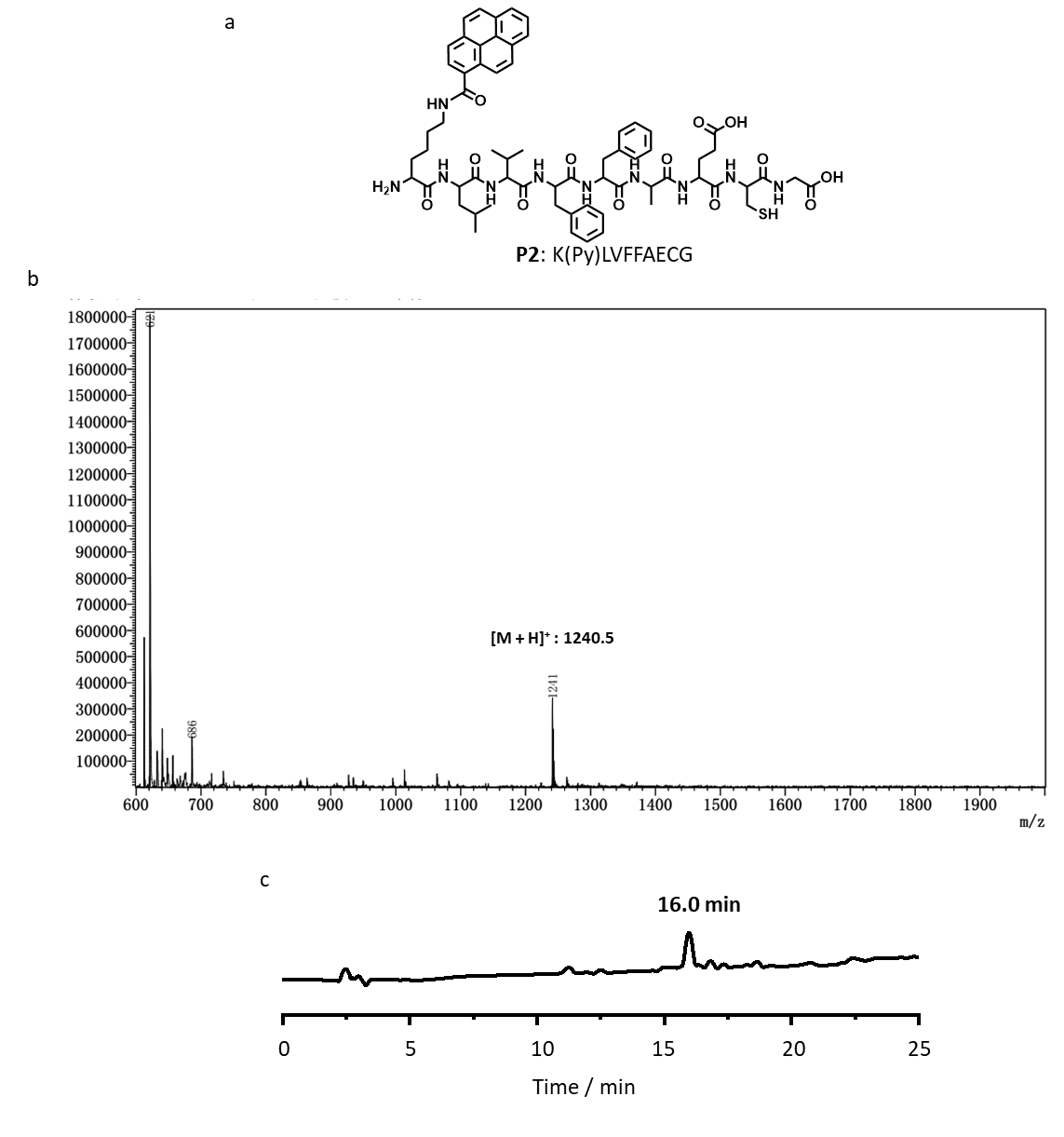
**

**Figure S7.** (a) Chemical structure of **P2**. (b) Electrospray Ionization (ESI) mass spectrometry analysis of **P2**. (c) HPLC analysis of **P2**.

**
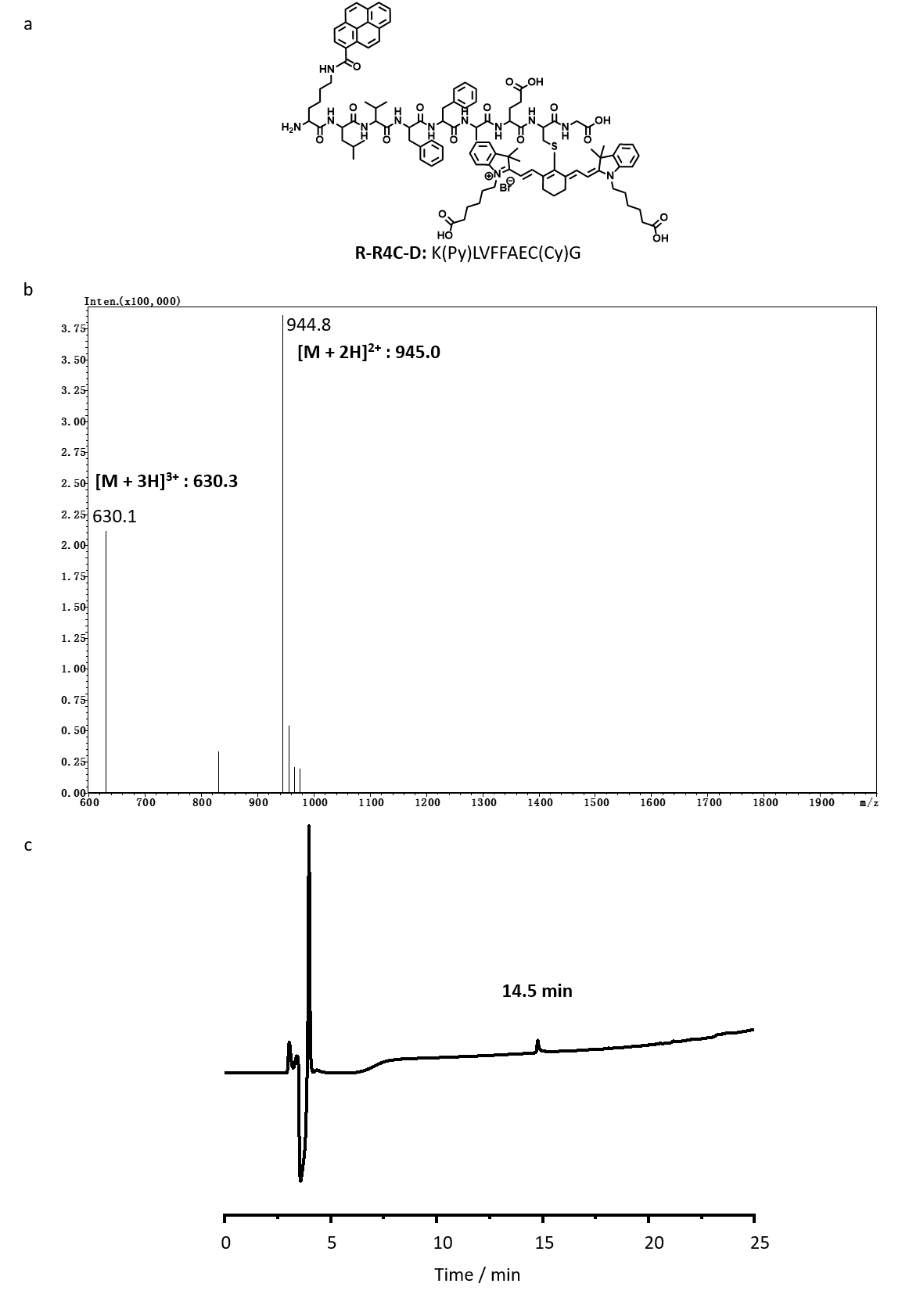
**

**Figure S8.** (a) Chemical structure of **R-R4C-D**. (b) Electrospray Ionization (ESI) mass spectrometry analysis of **R-R4C-D**. (c) HPLC analysis of **R-R4C-D**.

**
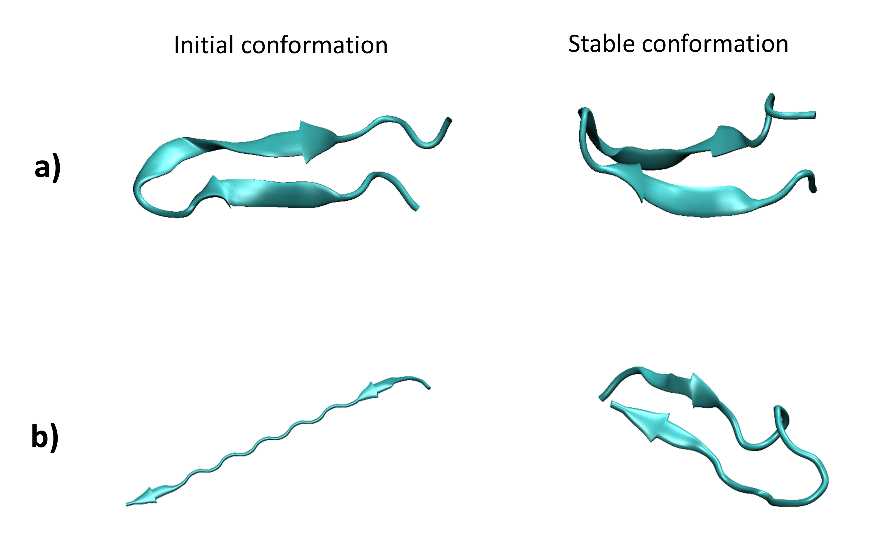
**

**Figure S9.** Comparison of stable conformations of **Pβ** with different initial conformations, I-TASSER (a) and straight line (b).

**
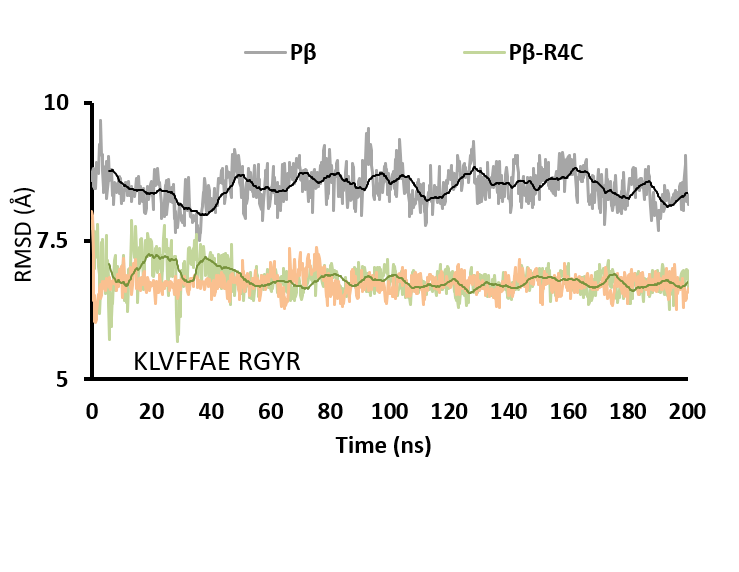
**

**Figure S10.** The radius of gyration (RGYR) comparison for assembly modules in **Pβ** and **Pβ-R4C**.

**
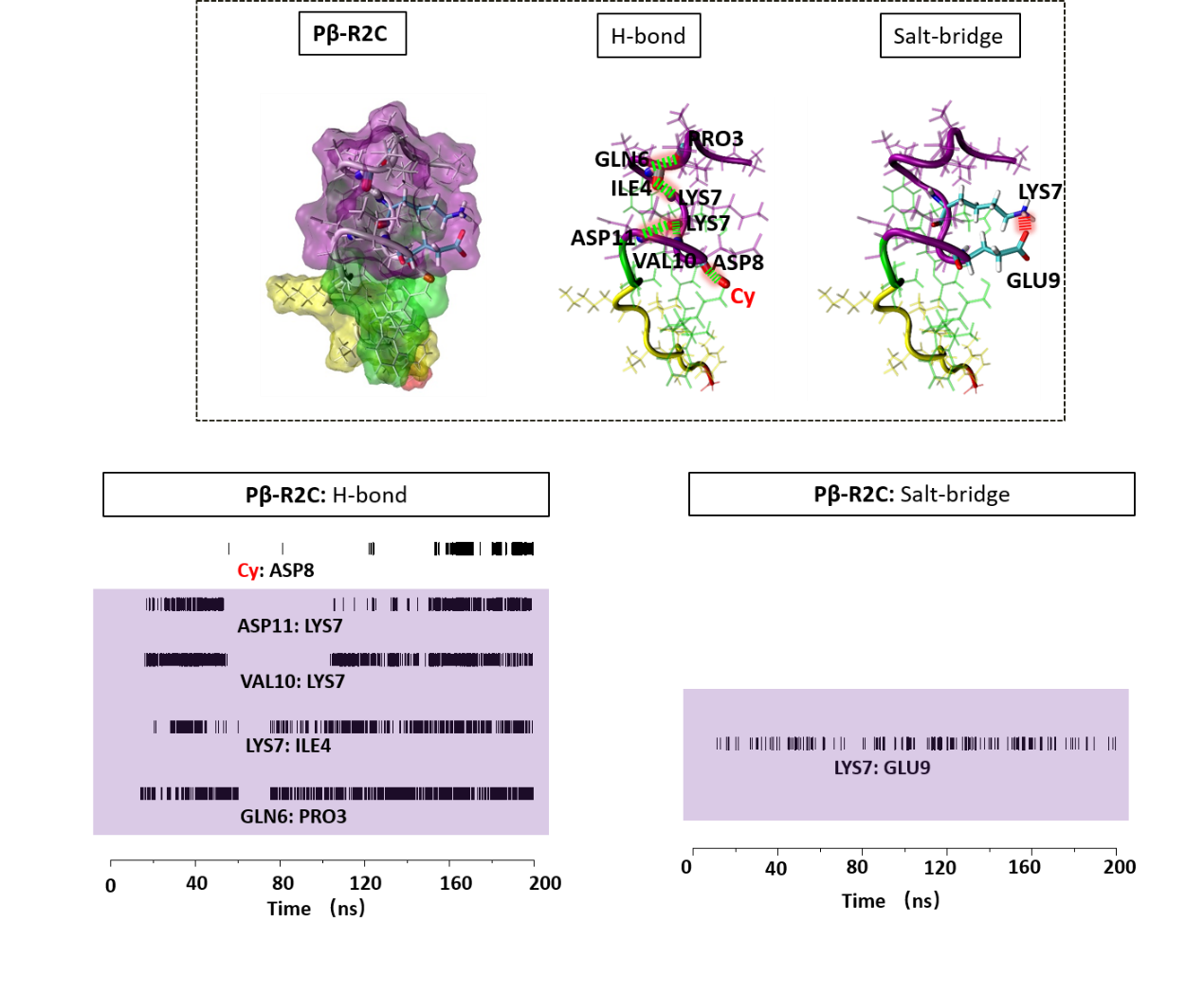
**

**Figure S11.** Molecular interaction details including hydrogen bond, salt-bridge and hydrophobic, together with the lifetime analysis of hydrogen bond and salt-bridge for **Pβ-R2C**.

**
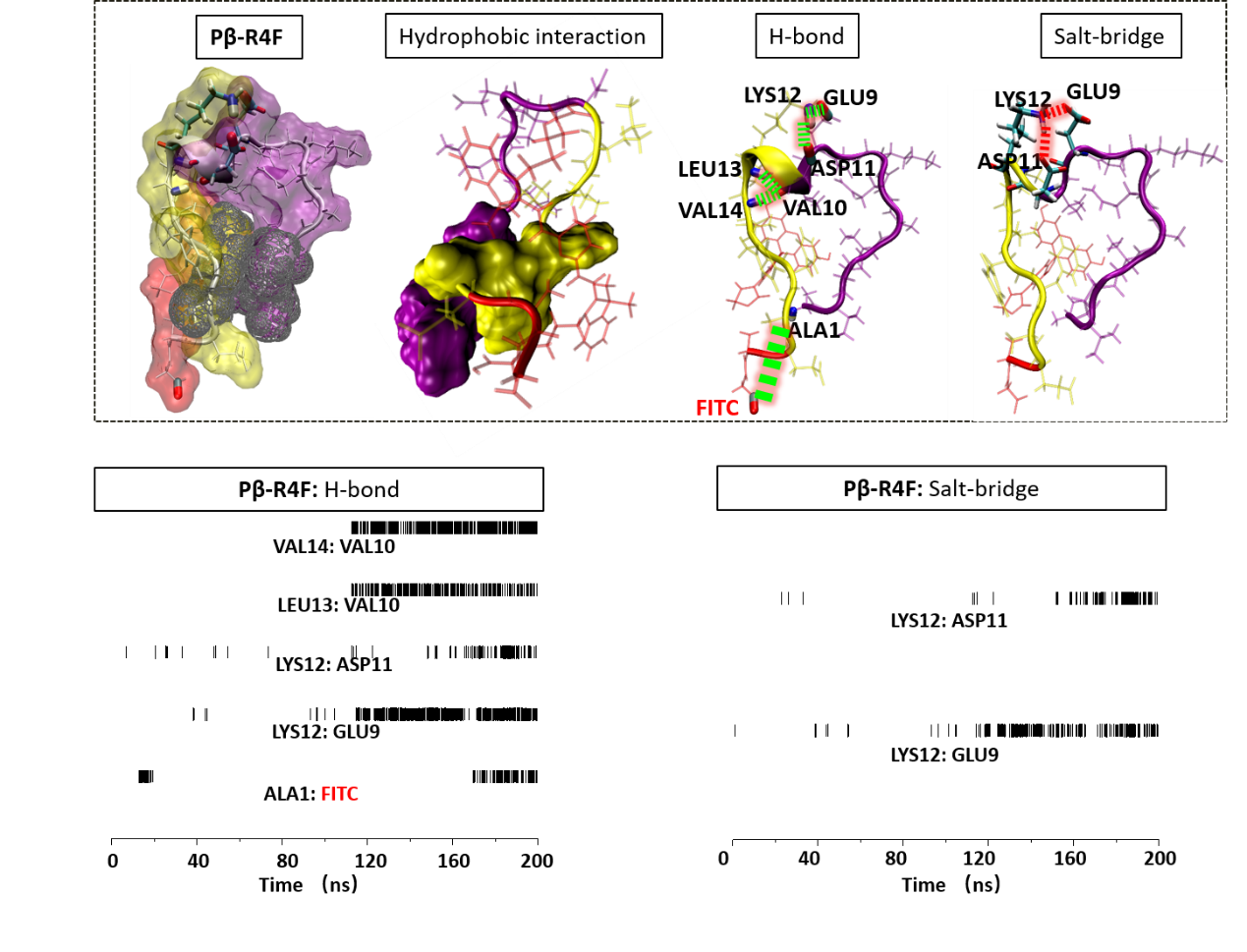
**

**Figure S12.** Molecular interaction details including hydrogen bond, salt-bridge and hydrophobic, together with the lifetime analysis of hydrogen bond and salt-bridge for **Pβ-R4F**.


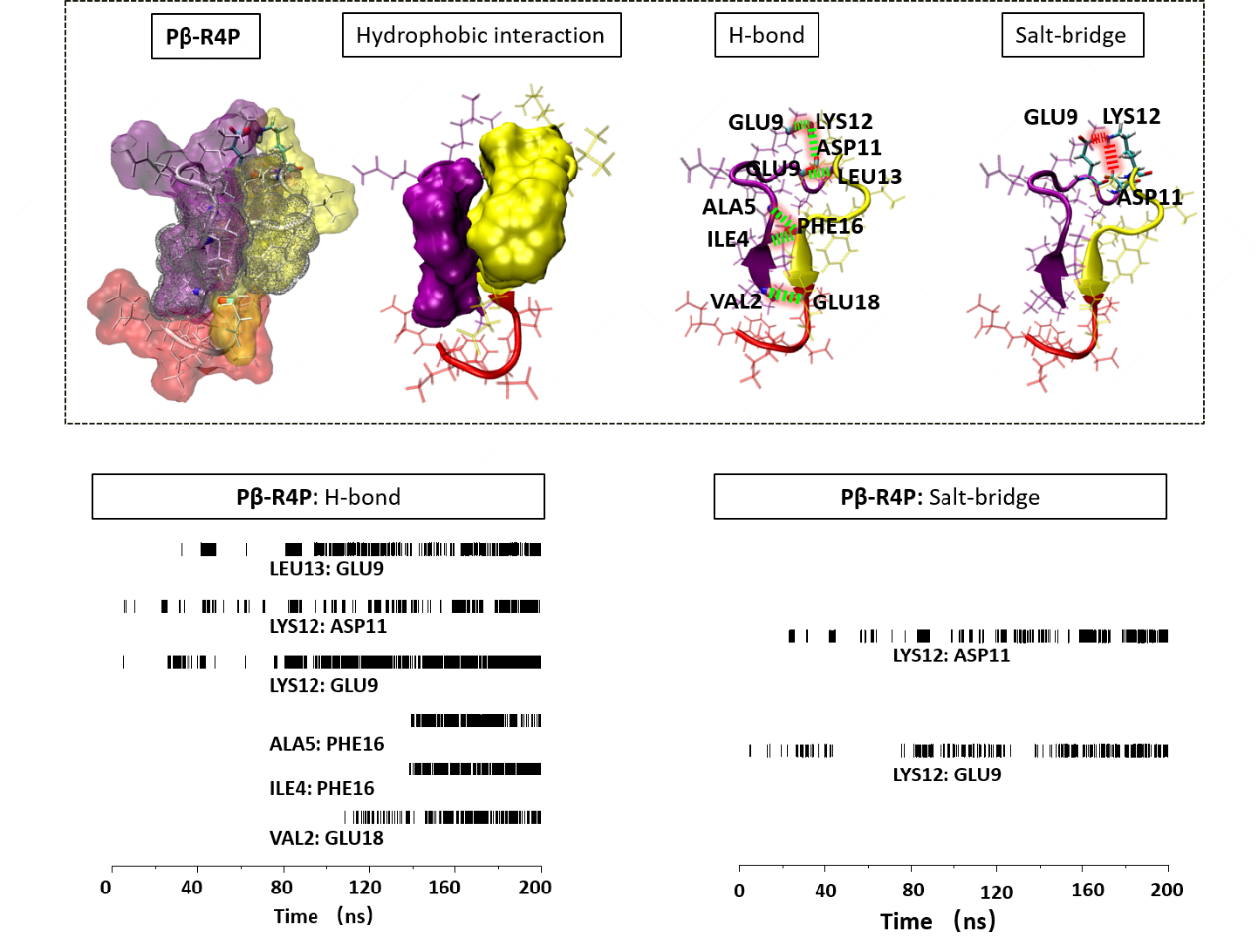


**Figure S13.** Molecular interaction details including hydrogen bond, salt-bridge and hydrophobic, together with the lifetime analysis of hydrogen bond and salt-bridge for **Pβ-R4P**.


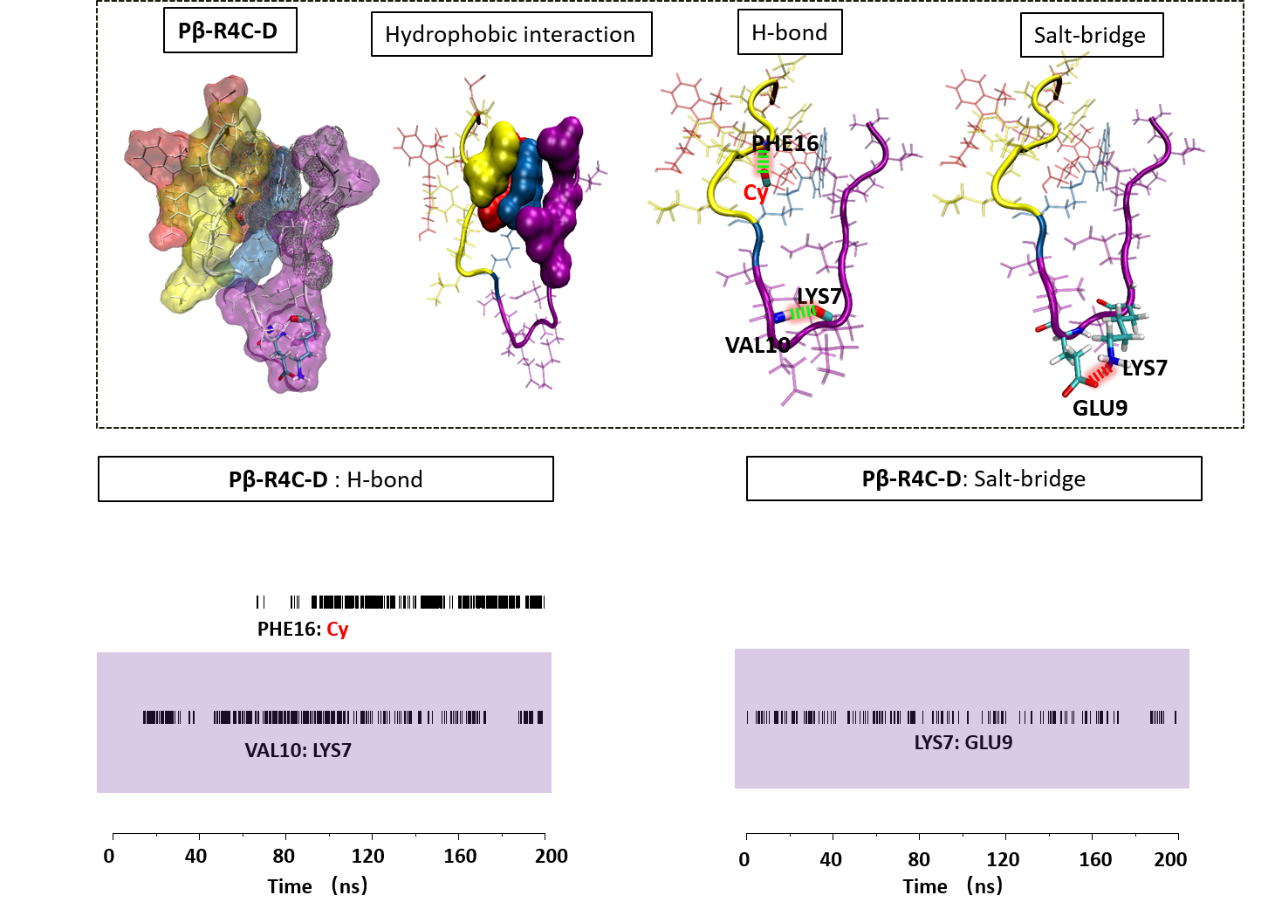


**Figure S14.** Molecular interaction details including hydrogen bond, salt-bridge and hydrophobic, together with the lifetime analysis of hydrogen bond and salt-bridge for **Pβ-R4C-D**.

**
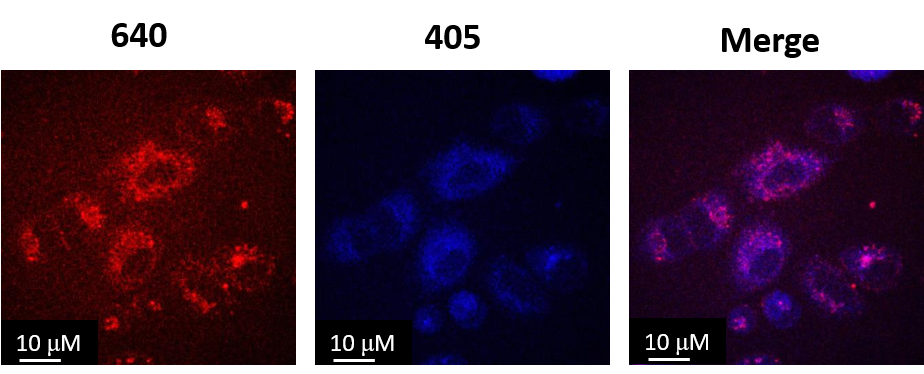
**

**Figure S15.** CLSM images of H460 coincubated with **Pβ-R4C-D**.

**Reference**

[1] H. W. An, S. L. Qiao, C. Y. Hou, Y. X. Lin, L. L. Li, H. Y. Xie, Y. Wang, L. Wang, H. Wang, *Chem Commun (Camb)* **2015**, *51*, 13488.

[2] Abraham, M. J.; Murtola, T.; Schulz, R.; Páll, S.; Smith, J. C.; Hess, B.; Lindahl, E.,*Softwarex* **2015**, *1-2*, 19.

[3] K. Lindorff-Larsen, S. Piana, K. Palmo, P. Maragakis, J. L. Klepeis, R. O. Dror, D. E. Shaw, *Proteins-structure Function & Bioinformatics* **2010**, *78*.

[4] W. L. Jorgensen, J. Chandrasekhar, J. D. Madura, R. W. Impey, M. L. Klein, *J. Chem. Phys.* **1983**, *79*.

[5] Hoover, William, Phys. Rev. A **1985**, *31*, 1695.

[6] M. Parrinello, A. Rahman, *J. Appl. Phys.* **1981**, *52*, 7182.

[7] S. Miyamoto, P. A. Kollman, *J. Comput. Chem.* **1992**, *13*, 952.

[8] T. Darden, D. York, L. Pedersen, *J. Chem. Phys.* **1993**, *98*, 10089.

[9] B. Hess, H. Bekker, H. J. C. Berendsen, J. Fraaije, *J. Comput. Chem.* **1997**, *18*, 1463.

[10] W. Humphrey, A. Dalke, K. Schulten, *J Mol Graph* **1996**, *14*, 33-38.

[11] Humphrey, W.; Dalke, A.; Schulten, K., *J Mol Graph* **1996,** *14*, 33
